# The draft genome of strain *c*Cpun from biting midges confirms insect *Cardinium* are not a monophyletic group and reveals a novel gene family expansion in a symbiont

**DOI:** 10.7717/peerj.6448

**Published:** 2019-02-21

**Authors:** Stefanos Siozios, Jack Pilgrim, Alistair C. Darby, Matthew Baylis, Gregory D.D. Hurst

**Affiliations:** 1 Institute of Integrative Biology, Faculty of Health and Life Sciences, University of Liverpool, Liverpool, UK; 2 Institute of Infection and Global Health, Faculty of Health and Life Sciences, University of Liverpool, Liverpool, UK; 3 NIHR Health Protection Research Unit in Emerging and Zoonotic Infections (HPRU-EZI), University of Liverpool, Liverpool, UK

**Keywords:** *Cardinium hertigii*, *Culicoides* biting midges, Genome sequence, Phylogenomic analysis, Gene family expansion, Heritable symbionts

## Abstract

**Background:**

It is estimated that 13% of arthropod species carry the heritable symbiont *Cardinium hertigii*. 16S rRNA and gyrB sequence divides this species into at least four groups (A–D), with the A group infecting a range of arthropods, the B group infecting nematode worms, the C group infecting *Culicoides* biting midges, and the D group associated with the marine copepod *Nitocra spinipes*. To date, genome sequence has only been available for strains from groups A and B, impeding general understanding of the evolutionary history of the radiation. We present a draft genome sequence for a C group *Cardinium*, motivated both by the paucity of genomic information outside of the A and B group, and the importance of *Culicoides* biting midge hosts as arbovirus vectors.

**Methods:**

We reconstructed the genome of *c*Cpun, a *Cardinium* strain from group C that naturally infects *Culicoides punctatus*, through Illumina sequencing of infected host specimens.

**Results:**

The draft genome presented has high completeness, with BUSCO scores comparable to closed group A *Cardinium* genomes. Phylogenomic analysis based on concatenated single copy core proteins do not support *Cardinium* from arthropod hosts as a monophyletic group, with nematode *Cardinium* strains nested within the two groups infecting arthropod hosts. Analysis of the genome of *c*Cpun revealed expansion of a variety of gene families classically considered important in symbiosis (e.g., ankyrin domain containing genes), and one set—characterized by DUF1703 domains—not previously associated with symbiotic lifestyle. This protein group encodes putative secreted nucleases, and the *c*Cpun genome carried at least 25 widely divergent paralogs, 24 of which shared a common ancestor in the C group. The genome revealed no evidence in support of B vitamin provisioning to its haematophagous host, and indeed suggests *Cardinium* may be a net importer of biotin.

**Discussion:**

These data indicate strains of *Cardinium* within nematodes cluster within *Cardinium* strains found in insects. The draft genome of *c*Cpun further produces new hypotheses as to the interaction of the symbiont with the midge host, in particular the biological role of DUF1703 nuclease proteins that are predicted as being secreted by *c*Cpun. In contrast, the coding content of this genome provides no support for a role for the symbiont in provisioning the host with B vitamins.

## Introduction

Invertebrates form a diverse range of symbiotic associations with heritable bacteria, microbes that pass from a female to her progeny. Ranging from less-intimate to highly specialized, these associations can confer novel phenotypic traits on their individual host, and thus may represent major drivers of both ecological and evolutionary dynamics (McLean et al., 2016; Sudakaran, Kost & Kaltenpoth, 2017; Ferrari & Vavre, 2011). Heritable bacteria can supplement the nutritionally imbalanced diet of hematophagous or sap feeding species with vitamins or essential amino acids, thus expanding the niche of the species (Rio, Attardo & Weiss, 2016; Hansen & Moran, 2014). Other symbionts exert protective effects against biotic or abiotic stress, including natural enemies (predators, parasitoids, fungi, bacteria, and viruses) (Brownlie & Johnson, 2009; Hansen, Vorburger & Moran, 2012) and heat stress (Dunbar et al., 2007). Notably, some heritable bacteria are parasitic and have evolved to manipulate host reproduction to increase the frequency of infected females and facilitate their own transmission (Hurst & Frost, 2015). These effects have further prompted their application in vector and pest management (Iturbe-Ormaetxe, Walker & O’ Neill, 2011).

*Cardinium* (Bacteroidetes) is a bacterial genus found in a wide range of arthropod species that has a wide variety of impacts on host individuals, including feminization (Weeks, Marec & Breeuwer, 2001; Groot & Breeuwer, 2006), parthenogenesis induction (Zchori-Fein et al., 2001), and cytoplasmic incompatibility (CI) (Hunter, Perlman & Kelly, 2003; Gotoh, Noda & Ito, 2006; Perlman, Kelly & Hunter, 2008; Ros & Breeuwer, 2009), alongside the capacity to improve host fitness (Weeks & Stouthamer, 2004). First discovered in 1996 (Kurtti et al., 1996), it is now estimated that c. 13% of arthropod species carry the symbiont (Weinert et al., 2015). *Cardinium* infections are found in a diverse set of arthropods, but its incidence is heterogeneous, with pronounced “hotspots” in arachnids (including spiders, mites, and harvestmen), diaspidid scale insects, parasitoid wasps, planthoppers, whiteflies, and biting midges (Duron et al., 2008; Zchori-Fein & Perlman, 2004; Gruwell, Wu & Normark, 2009; Nakamura et al., 2009; Chang et al., 2010; Morag et al., 2012; Lewis et al., 2014; Mee et al., 2015). Further symbioses are observed with plant parasitic nematodes (Noel & Atibalentja, 2006; Denver et al., 2016), copepods (Edlund et al., 2012), non-marine ostracods (Schön et al., 2018), and oribatid mites (Konecka & Olszanowski, 2018) suggesting that the true diversity of the genus is yet to be appreciated. This wider clade *Cardinium* represents the sister group to the amoeba symbiont *Amoebophilus asiaticus* (Nakamura et al., 2009; Schmitz-Esser et al., 2010; Santos-Garcia et al., 2014).

Phylogenetic analyses of *Cardinium* based on two gene sequences (*16S* rRNA and *gyr*B) inferred the existence of at least four monophyletic groups designated as A, B, C, and D (Nakamura et al., 2009; Edlund et al., 2012), resembling *Wolbachia* super-groups in terms of host-affinities (Lo et al., 2002). Group A is the largest and the most studied of the three groups and has been found in various arthropod species. Group B has been found in plant parasitic nematodes (Noel & Atibalentja, 2006; Denver et al., 2016) and is represented by *Cardinium* strains *c*HgTN10, an endosymbiont of the soybean cyst nematode *Heterodera glycines* (Noel & Atibalentja, 2006) and *c*Ppe, an endosymbiont of the plant parasitic nematode *Pratylenchus penetrans* (Brown et al., 2018). Group C consists of a phylogenetically distinct clade of *Cardinium* strains known only from species of *Culicoides* biting midges, an important group of hematophagous pests and vectors of arboviruses and parasites (Nakamura et al., 2009; Morag et al., 2012; Lewis et al., 2014; Mee et al., 2015). Finally, group D have been found as a constituent of the bacterial communities associated with the marine copepod *Nitocra spinipes* (Edlund et al., 2012).

To date, genomic characterization has been restricted to A and B group *Cardinium* strains. Three insect-associated A-group *Cardinium* strains have been sequenced. These include the CI-inducing *Cardinium* endosymbiont (*c*Eper1) of the parasitic wasp *Encarsia pergandiella* (Penz et al., 2012), the *Cardinium* endosymbiont (*c*BtQ1) of the whitefly *Bemisia tabaci* (Santos-Garcia et al., 2014) and the *Cardinium* endosymbiont (*c*Sfur) of the planthopper *Sogatella furcifera* (Zeng et al., 2018). These genome sequences have indicated that convergent phenotypes, like CI, have a divergent genetic basis in *Cardinium* from *Wolbachia*. Moreover, the *c*Eper1 *Cardinium* genome suggests the symbiont may supplement B-vitamin provision (Penz et al., 2012), a phenotype that would be important in bloodsucking vectors. More recently, the genome sequences for two B group *Cardinium* strains from nematodes have been completed. These are the genomes of the *Cardinium* endosymbiont (*c*HgTN10) from *H. glycines* (Showmaker et al., 2018) and the *Cardinium* endosymbiont *c*Ppe from *P. penetrans* (Brown et al., 2018). However, there is no available genome for the C clade *Cardinium,* which is particularly notable in the light of the pest and vector status of the host species.

In this paper, we present an annotated draft genome sequence for a *Cardinium* endosymbiont from clade C, carried by the biting midge *Culicoides punctatus*, hereafter *c*Cpun, and use this genome data to estimate the relationship between C clade *Cardinium* and those of A and B groups; improving our understanding of strain relatedness that currently rest on the sequence of two loci. We further use the genome sequence to infer potential aspects of the symbiosis between this microbe and *Culicoides* biting midges. The study of midge symbionts is important, as the symbiosis may potentially have an impact on the physiology of a bloodsucking host, and (by parallel with *Wolbachia*) its vector competence for arboviruses and other pathogens. The difficulty of growing midges in insectary culture has presented a challenge to determining the effect of the symbiont on the host experimentally. Analysis of the *c*Cpun genome and comparison to the previously sequenced *Cardinium* genomes as well as their sister species *A. asiaticus* (Schmitz-Esser et al., 2010) was therefore undertaken to provide insight into the evolution and life style of clade C *Cardinium*.

## Materials and Methods

### Genome sequencing, assembly, and annotation

*Culicoides punctatus* female midges were collected from Leahurst Campus, University of Liverpool, UK using UV light traps and identified from wing morphology and by cytochrome c oxidase subunit 1 barcoding as in Pilgrim et al. (2017). DNA was extracted from single individuals using the QIAGEN DNAeasy™ Blood & Tissue Kit following the protocol for purification of total DNA from Insect. All samples were tested for *Cardinium* infection using a PCR assay based on 16S rRNA *Cardinium* specific primers Car-sp-F 5′-CGGCTTATTAAGTCAGTTGTGAAATCCTAG-3′; Car-sp-R 5′-TCCTTCCTCCCGCTTACACG-3′ (Nakamura et al., 2009). Whole-genome sequencing was carried out by the Centre for Genomic Research, University of Liverpool using the Illumina TruSeq Nano library preparation protocol. Two short-insert (∼550 bp insert size) paired-end libraries were constructed from two pooled DNA samples of three individuals each. The libraries were multiplexed and sequenced using 2/3 of a lane on an Illumina HiSeq 2500 platform, yielding 2 × 125 bp paired reads. Adapter removal and quality trimming of the raw Illumina reads were performed with Cutadapt version1.2.1 (Martin, 2011) and Sickle version 1.2 (Joshi & Fass, 2011).

Identification and filtering of symbiont reads were performed using a similar approach to that used previously (Pilgrim et al., 2017). Briefly, a preliminary assembly of the quality trimmed dataset was performed using SPAdes version 3.7.0 (Nurk et al., 2013) using the following parameters (-k 21,33,55,77, –careful, –cov-cutoff 5). The initial contigs were visualized using taxon-annotated GC-coverage plots (Fig. S1) with Blobtools (Kumar et al., 2013; Laetsch, 2016). Additional tblastx searches (Altschul et al., 1997; Camacho et al., 2009) were conducted against a local genomic database consisting of *Cardinium* genomes—*c*BtQ1 and *c*Eper1 endosymbionts of the whitefly *B. tabaci* and the parasitic wasp *E. pergandiella*, respectively (Santos-Garcia et al., 2014; Penz et al., 2012), that of *Cardinium* strain *c*HgTN10 from *H. glycines* (Showmaker et al., 2018) and the more distantly related *Acanthamoeba* endosymbiont *A. asiaticus* (Schmitz-Esser et al., 2010)—with an *e*-value cut-off of 1*e*^−^^6^. *Cardinium* contigs were extracted and checked for contamination by blastx searches against the non-redundant (nr) protein database. *Cardinium*-specific reads were subsequently retrieved using Bowtie2 (Langmead & Salzberg, 2012) and samtools (Li et al., 2009) and re-assembled de novo using SPAdes as described above. All contigs larger than 500 bp were checked for potential host or other bacteria contamination using blastx searches against nr database and all contaminant contigs were removed from the final assembly. Subsequently, we evaluated the quality of the assembled contigs using the reference-free assembly validation tool REAPR (Hunt et al., 2013). REAPR uses read pairs mapping information to identify potential assembly errors and assign quality scores on each base of the assembly. The error calls were then used to break the pre-assembled contigs at every potential miss-assembly position using the aggressive option “-a.” Finally, the broken assembly was scaffolded using SSPACE (Boetzer et al., 2011) using the default parameters.

The *c*Cpun draft genome was annotated using Prokka version 1.12 (Seemann, 2014) and the completeness was assessed using BUSCO v3 based on the presence of 148 universal bacterial marker genes (Simão et al., 2015). Clusters of Orthologous Groups (COG) functional categories were assigned using the eggNOG database (Huerta-Cepas et al., 2016) while additional domains were assigned by searches against the Pfam protein database (Finn et al., 2016). The k-mer fraction of the filtered reads were computed with Jellyfish v2.2.3 (Marçais & Kingsford, 2011) and used to determine the repeat fraction of *c*Cpun genome using GenomeScope (Vurture et al., 2017). Finally, comparison of the repeat density (repeats ≥ 200 bp and at least 95% identity) between the Amoebophilaceae genomes was performed using MUMmer-plots (Kurtz et al., 2004).

### Ortholog identification, comparative, and phylogenetic analyses

The genome sequences of the three available arthropod-associated *Cardinium* strains *Cardinium hertigii c*Eper1 (Penz et al., 2012), *Cardinium hertigii c*BtQ1 (Santos-Garcia et al., 2014) and *Cardinium c*Sfur (Zeng et al., 2018), the two nematode-associated endosymbionts *c*HgTN10 and *c*Ppe (Showmaker et al., 2018; Brown et al., 2018) and the *Acanthamoeba* endosymbiont *A. asiaticus* (Schmitz-Esser et al., 2010) were obtained from GenBank and used for comparative analyses (accession numbers GCF_000304455.1, GCF_000689375.1, GCA_003351905.1, GCA_003176915.1, and GCF_000020565.1, respectively). The genomes of *Cyclobacterium marinum* DSM 745 (GCF_000222485.1) and *Marivirga tractuosa* DSM 4126 (GCF_000183425.1), two free living *Bacteroides* species, were used as outgroup for the phylogenetic analyses (based on Santos-Garcia et al., 2014). All GenBank retrieved genomes were re-annotated using Prokka software as described above in order to mitigate the effect of inconsistencies due to alternative annotation practices. Orthologous groups of proteins were identified between *c*Cpun, *c*Eper1, *c*BtQ1, *c*Sfur, *c*HgTN10, *c*Ppe, and *A. asiaticus* using an all-vs-all BLAST search and Markov Cluster (MCL) clustering approach as implemented in OrthoFinder method (Emms & Kelly, 2015). Core, accessory and strain-specific orthogroups between the five genomes were visualized with an UpSet plot using the UpSetR package (Conway et al., 2017).

Phylogenetic reconstruction was performed on a set of 278 single copy core protein sequences shared between the six *Cardinium* genomes, the genome of *A. asiaticus* and two free living *Bacteroides* species (*Cyclobacterium marinum* and *M. tractuosa*) that were used as outgroup. To this end, a super-matrix was generated by concatenating the protein alignments of the 278 core proteins and trimmed with trimAl version 1.4 (Capella-Gutiérrez, Silla-Martínez & Gabaldón, 2009) using the “automated” option. The best substitution model (LG+F+R4) was selected using ModelFinder (Kalyaanamoorthy et al., 2017) and phylogenetic inference was performed using the maximum likelihood (ML) criterion as implemented in IQ-TREE v1.6.6 (Nguyen et al., 2015). The robustness of the inferred tree was finally assessed with the ultrafast bootstrap approximation method as implemented in IQ-TREE using 1,000 replicates (Hoang et al., 2018). Alternative phylogenetic hypotheses were tested by constrained tree searches using the approximately unbiased (AU) test (Shimodaira & Goldman, 2002) as implemented in IQ-TREE v1.6.6. Additionally, the distribution of the phylogenetic signal across the concatenated super-matrix was calculated as described in (Shen, Hittinger & Rokas, 2017). Briefly, for each of the 278 core protein alignments the log-likelihood score for the best ML tree topology under concatenation and an alternative conflicting topology was calculated under the same substitution model (LG+F+R4). The difference in the gene-wise log-likelihood scores (ΔGLS) between the two alternative topologies was used as a measure of the phylogenetic signal and to visualize the proportion of core genes supporting each conflicting phylogeny. Finally, an independent phylogenetic analysis was performed on a subset of 46 core ribosomal proteins in IQ-TREE v1.6.6 as described above in order to further test the robustness of our phylogenetic inference. Phylogenetic trees were drawn and annotated online using the EvolView tool (He et al., 2016).

### Analyses of the DUF1703 gene family expansion

Genome analysis revealed an expansion of the DUF1703 gene family. To analyze this expansion further, a protein sequence alignment of the DUF1703 gene family from *Cardinium* together with selected Open Reading Frames (ORFs) with sequence similarity retrieved as best BLAST hits form NCBI’s nr database was performed using MAFFT v7 and default parameters (Katoh & Standley, 2013). Ambiguously aligned positions were subsequently removed using trimAl version 1.4 and the “automated” option. A ML phylogenetic analyses was performed with IQ-TREE version 1.6.6 and the phylogenetic tree were constructed and annotated as described above. Additionally, a neighbor-net phylogenetic network was inferred from the translated nucleotide alignment of the *c*Cpun DUF1703 paralogs using SplitsTree version 4.12.6 (Huson & Bryant, 2006; Bryant & Moulton, 2004) and default parameters. A pairwise identity and similarity matrix of the *c*Cpun DUF1703 amino acid sequence paralogs were constructed using the Needleman–Wunsch global alignment method and the BLOSUM62 substitution matrix as implemented in EMBOSS package (Rice, Longden & Bleasby, 2000). Putative signal peptides were predicted on the SignalP 4.1 Server (Petersen et al., 2011) using the sensitive D-cutoff settings. Detection of putative recombination events was performed using the RDP4 software package (Martin et al., 2015). Recombination Detection Program (RDP) implements several methods for detecting recombination signals including MaxChi (Smith, 1992), GENECONV (Padidam, Sawyer & Fauquet, 1999), BottScan (Salminen et al., 1995), Chimera (Posada & Crandall, 2001), and RDP (Martin & Rybicki, 2000). Global parameters were as follow: *P*-value cutoff was set to 0.001 using a Bonferroni correction and significance was evaluated from a permutation test based on 1,000 permutations. Detected signals were considered significant only when they were confirmed by multiple methods. Inference of recombination signals can be particularly misleading when diverse sequences are analyzed. To avoid such misalignment artefacts, the 25 complete DUF1703 paralogs were grouped into three groups on the bases of nucleotide sequences similarity (>65%) and the analyses was repeated for each group separately. Finally, the results were also confirmed with PhiPack implementing the pairwise homoplasy index (PHI) algorithm (Bruen, Philippe & Bryant, 2006). Residue composition and conservation within the core nuclease PD-(D/E)XK site of the DUF1703 homologs were illustrated with sequence logos using the Skylign tool (Wheeler, Clements & Finn, 2014).

### Nucleotide sequence accession numbers

The raw reads and the *c*Cpun draft genome assembly have been submitted to the DDBJ/EMBL/GenBank database under the BioProject accession number PRJNA487198 (WGS project QWJI00000000).

## Results and Discussion

### General features of *c*Cpun draft genomes

The final assembly of the *c*Cpun draft genome consists of 57 scaffolds larger than 500 bp (N50 = 41.6 kb, largest scaffold = 116 kb) comprising a total size of 1,137,634 bp (52 scaffolds ≥ 1,000 bp) with an average GC content of ∼33% and an average depth of coverage 90× (Table 1; Fig. S2). Overall, the *c*Cpun genome shares many characteristics with those of the previously sequenced *Cardinium* strains *c*Eper1, *c*BtQ1, *c*Sfur, cHgTN10, and *c*Ppe including similar genome size of around one Mb and comparable GC content (33.7–38%) (Table 1). No plasmids were inferred based on the presence of scaffolds with atypically higher read coverage compared with the average coverage of the complete assembly, presenting a contrast to the previously sequenced arthropod-associated *Cardinium* (*c*Eper1 and *c*BtQ1) (Table 1; Fig. S2). Nevertheless, we were able to detect several regions with sequence similarity to elements of the two plasmids found in *c*Eper1 and *c*BtQ1. Matching regions were mainly transposases, suggesting that these might be remnants of ancestral plasmid invasion/s. Although absence of plasmids has also been reported previously for *A. asiaticus,* the sister species of *Cardinium* clade (Schmitz-Esser et al., 2010), the presence of low-copy-number plasmids in *c*Cpun cannot be ruled out.

**Table 1:** Genome Features of *c*Cpun draft genome and its closest relatives.

	*c*Cpun	*c*Eper1**	*c*BtQ1**	*c*Sfur	*c*HgTN10	*c*Ppe	*A. asiaticus*
Number of scaffolds	57*	1	11	1	1	27	1
Plasmids	0	1	1	0	0	0	0
Total size in kb	1,137	887 (58)	1,013 (52)	1,103	1,193	1,358	1,884
GC content (%)	33.7	36.6 (31.5)	35 (32)	39.2	38.2	35.8	35
CDS	917	841 (65)	709 (30)	795	974	1,131	1,557
Avg. CDS length (bp)	993	911 (733)	1,033 (1,389)	1,052	997	941	990
Coding density (%)	80	85.5 (82.1)	79.7 (80.1)	75.7	81.4	78.3	81.8
rRNAs	3	3	3	3	3	3	3
tRNAs	37	37	35	35	37	34	35
Ankyrin repeat proteins	46	18-19	26	29	27	32	54
Reference	this study	a	b	c	d	e	f

**Notes:**

a Penz et al. (2012).

b Santos-Garcia et al. (2014).

c Zeng et al. (2018).

d Showmaker et al. (2018).

e Brown et al. (2018).

f Schmitz-Esser et al. (2010).

* contigs > 500 bp.

** chromosome (plasmid).

A total of 917 protein coding genes were identified with an average length of 993 bp corresponding to a coding density of around 80% (Table 1; Table S1). *c*Cpun harbors a single set of rRNA genes with the 16S separated from 5S and 23S and encode a complete set of 37 tRNA genes. The identification of 117 out of the 148 BUSCO marker genes (BUSCO score = C: 79% (S: 79%, D: 0%), F: 2.7%, M: 18.2%, *n*: 148) (Fig. S3) was comparable to that observed for the previously sequenced and complete *c*Eper1 *c*Sfur and cHgTN10 genomes, which suggests that *c*Cpun is a near complete genome. Overall, the redundancy in *c*Cpun as assessed through MUMmer-plots is lower than both *A. asiaticus* and *c*BtQ1 previously described as highly repetitive (Santos-Garcia et al., 2014) (Fig. S4). K-mer frequency analysis of the Illumina reads estimated the repetitive fraction of *c*Cpun genome to be circa 13%.

### Phylogenomic analyses place *c*Cpun as an outgroup of both other insect and nematode *Cardinium* strains

Recently, a new family named Amoebophilaceae was proposed to include the *Cardinium* clades as well as the amoeba-associated *A. asiaticus* (Santos-Garcia et al., 2014). Currently, at least four major phylogenetic clades of *Cardinium* related bacteria have been described (Nakamura et al., 2009; Edlund et al., 2012) with possible evidence for additional clades (Chang et al., 2010). However, the phylogenetic (evolutionary) relationships between these clades are not clear. Previous phylogenetic studies based on partial 16S rRNA and gyrB sequences failed to provide a consistent phylogenetic placement for the arthropod and the nematode *Cardinium* clades (Morag et al., 2012; Nakamura et al., 2009).

**Figure 1:**
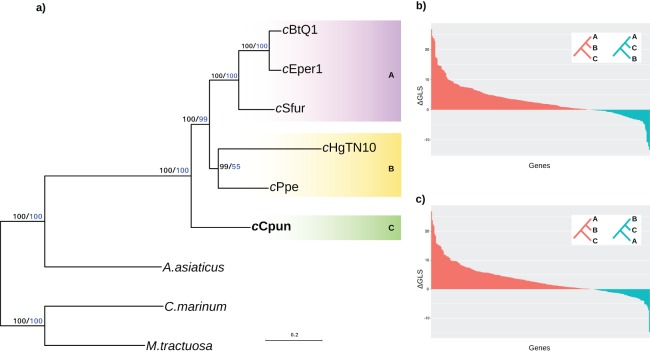
Phylogenetic relationships of *Cardinium* strains. (A) The phylogenetic tree was inferred from the concatenated analysis of 278 single copy core proteins and separately from a subset of 46 core ribosomal proteins using the Maximum Likelihood method as implemented in IQTRE v1.6.6 (model: LG+F+R4). Both datasets retrieved the same tree topology and here we present only the first one. The numbers on the branches represent support values based on 1,000 bootstrap replicates (black bold values: complete matrix; blue values: ribosomal dataset). The three major *Cardinium* groups A, B, and C are denoted with different color shading. *Cyclobacterium marinum* and *Marivirga tractuosa*, two free living members of Bacteroidetes were used as outgroups. (B, C) Distribution of the phylogenetic signal in *Cardinium* concatenated ML phylogeny. The gene-wise differences in log-likelihood scores (ΔGLS) between the concatenated Maximum likelihood tree in (A) versus two alternative topologies: A,C-groups monophyletic relative to B-group (B) and B,C-groups monophyletic relative to A-group (C) were calculated as described in (Shen, Hittinger & Rokas, 2017) and plotted in descending order. The red bars represent the genes supporting the Maximum likelihood tree while the blue bars represent the genes supporting each of the alternative topologies.

We established the relationship of this group across a concatenated set of 278 single copy core protein coding genes as well as a subset of 46 ribosomal protein genes shared between the seven Amoebophilaceae genomes. The results of both analyses clearly support the position of the midge *Cardinium* clade (C) as a sister group to both the other arthropod and nematode *Cardinium* clades (clades A and B) and confirm that the arthropod-associated *Cardinium* do not form a monophyletic group (Fig. 1A). Constrained tree tests for two alternative topologies (a) nematode *Cardinium* as sister group of all other arthropod *Cardinium* and (b) *c*Cpun and nematode *Cardinium* as a monophyletic group resulted in significantly worse trees (AU test, *p* < 0.01). This inference was further supported by analysis of single protein phylogenies (Figs. 1B and 1C). A total of 157 out of the 278 single copy core genes (56%) support the monophyletic grouping of the B group *Cardinium* strains (*c*HgTN10, *c*Ppe) with the A group (*c*Eper1, *c*BtQ1 and *c*Sfur) in exclusion of *c*Cpun (*p* < 0.001, Fisher’s exact test). In contrast, only 68 genes (24%) support the monophyletic grouping of *c*Cpun with the A group strains while a small subset of genes (*n* = 52; 19%) supports the monophyletic grouping of *c*Cpun with *c*HgTN10 and *c*Ppe.

### Genome content comparisons estimate both a core *Cardinium* genome, genes associated with an insect-symbiont lifestyle, and *c*Cpun specific genes and gene families

**Figure 2:**
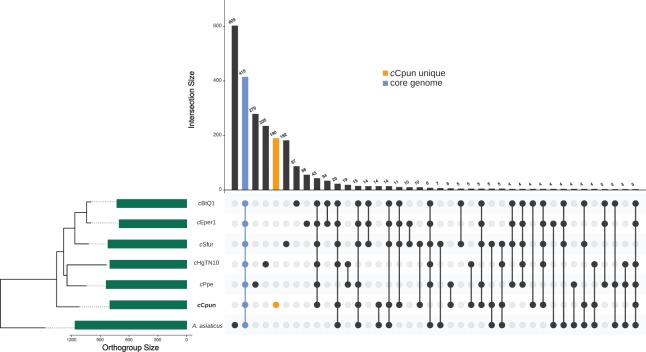
Genome content comparison across the seven *Amoebophilaceae* genomes. UpSet plot showing unique and overlapping protein ortholog clusters across the seven Amoebophilaceae genomes *c*Cpun, *c*Eper1, *c*BtQ1, *c*Sfur, *c*HgTN10, *c*Ppe, and *Amoebophilus asiaticus*. The intersection matrix is sorted in descending order. Green bars represent the orthogroup size for each genome ordered by their phylogenetic relationships. Connected dots represent intersections of overlapping orthogroups while vertical bars shows the size of each intersection. The core orthogroup and the *c*Cpun unique orthogroup cluster are shown with the blue and the orange bars respectively. The plot was generated using UpSetR package in R (Conway et al., 2017).

The OrthoFinder clustering algorithm identified a total of 2,015 ortholog protein clusters across the seven Amoebophilaceae genomes (*A. asiaticus*, *c*HgTN10, *c*Ppe, *c*Cpun, *c*Eper1, *c*Sfur, and *c*BtQ1). The seven genomes share a core of 415 ortholog clusters of which 278 consist of single-copy genes (Fig. 2). The *c*Cpun genome codes for a substantial number of unique proteins (Fig. 2; Table S2). Specifically, among the 812 ortholog clusters predicted for *c*Cpun, 190 clusters—including 204 protein coding genes—were assigned as strain-specific (Fig. 2). Of these genes, 40 were predicted to code for proteins of less than 70 amino acids and likely represent either annotation artefacts or pseudogenised gene fragments.

The majority of *c*Cpun specific proteins, 138 (∼67%), had neither significant matches (blastp, *e*-value ≤ 10^−10^) in the NCBI-nr database, nor predicted functional domain. These were assigned as hypothetical proteins. Amongst the remaining 66 predicted *c*Cpun-specific protein clusters, those with ankyrin-repeat domains were particularly well represented in the strain specific set (Table S2). The abundance, diversity and presumably eukaryotic origin ANK repeat containing proteins has long led them to be considered likely to be involved in symbiotic interactions, and this has been demonstrated in a few cases (Siozios et al., 2013; Nguyen, Liu & Thomas, 2014; Voth, 2011; Pan et al., 2008). A total of 46 ANK repeat proteins were present in the *c*Cpun genome, which represents the largest expansion of this gene family in *Cardinium*, comparable to the expansion of this family in *A. asiaticus* (54 ANK proteins) (Schmitz-Esser et al., 2010). In total, 20 out of the 46 ankyrin repeat-containing proteins identified in *c*Cpun were not found in the other *Cardinium* strains, suggesting potential host-specific functions. Among the remaining strain-specific protein clusters, 13 were assigned as putative mobile elements (transposases), three putative transporters, a DNA repair protein RecN, two putative GNAT-family acetyltransferases and a homologue of the hemolysin transporter protein ShlB (Table S2). Finally, a folylpolyglutamate synthase (FolC) homologue involved in the tetrahydrofolylpolyglutamate biosynthesis pathway and a putative riboflavin biosynthesis protein RibBA were also detected. Absence of the complete pathway for the de novo biosynthesis of folate in *c*Cpun suggest that FolC probably participates in the folate salvage pathway (folate to polyglutamate) as suggested also by the presence of a dihydrofolate reductase homologue (De Crécy-Lagard et al., 2007).

Candidate proteins related to the adaptation of *Cardinium* to arthropod hosts (as opposed to Amoeba and nematode) were identified as being in the four arthropod-associated *Cardinium* strains (*c*Cpun, *c*Sfur, *c*Eper1, and *c*BtQ1), and not *Amoebophilus* and the nematode-associated *Cardinium* strains (*c*HgTN10 and *c*Ppe). The four strains from whitefly, wasp, planthopper and midge uniquely share 11 ortholog protein clusters (Fig. 2). Among them we observed the virulence-associated E family protein previously detected in the plasmids harbored by *c*Eper1 and *c*BtQ1 (Penz et al., 2012; Santos-Garcia et al., 2014) and a nicotinamide mononucleotide transporter.

### *c*Cpun possesses both afp-like and type IX secretion systems

**Figure 3:**
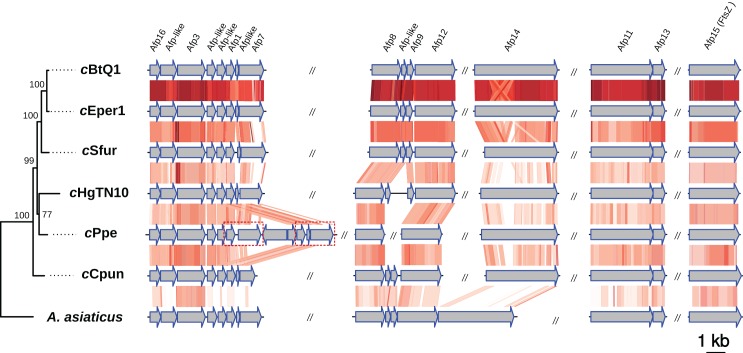
Organization and comparison of the antifeeding prophage (Afp-like) genes clusters in the seven Amoebophilaceae genomes. The phylogeny of the Afp-like secretion system was inferred with Maximum Likelihood based on the concatenated alignment of the 15 constituent protein sequences using IQTREE v1.6.6. Conserved regions are connected with a gradient of red shadings based on tblastx identities. The dash-line rectangles denote a duplicated region in *c*Ppe strain described in Brown et al. (2018). The synteny and the phylogenetic tree of the Afp-like gene clusters were visualized using the genoPlotR package (Guy, Roat Kultima & Andersson, 2010).

Intracellular microbes utilize a variety of specialized protein secretion systems in order to invade and interact with their eukaryote host (Tseng, Tyler & Setubal, 2009; Dale & Moran, 2006). A common characteristic of the Amoebophilaceae genomes is that all encode for a putative afp-like protein secretion system presumably involved in host-microbe interactions (Penz, Horn & Schmitz-Esser, 2010; Penz et al., 2012; Hurst et al., 2007). This system was also observed in the *c*Cpun genome (Fig. 3) (Penz, Horn & Schmitz-Esser, 2010; Penz et al., 2012; Santos-Garcia et al., 2014). The organization of the AFP-like genes clusters is conserved between the four Amoebophilaceae genomes and suggests operon-like structures (Fig. 3).

We additionally identified seven components of the type IX secretion system (T9SS) in *c*Cpun, a system related to gliding motility and pathogenicity in several members of the phylum *Bacteroidetes* (McBride & Zhu, 2013; McBride & Nakane, 2015). *c*Cpun is the third *Cardinium* strain reported to retain components of the T9SS system (Santos-Garcia et al., 2014; Zeng et al., 2018). Four of these protein clusters with homology to the core components of the T9SS (GldK, GldL, GldM, GldN) are shared between *c*Cpun, *A. asiaticus*, *c*BtQ1, and *c*Sfur while an additional three proteins with homology to the lipoproteins GldD, GldJ, and GldH are uniquely shared between *c*Cpun and *A. asiaticus* with exception the GldJ which was also found in the *c*Sfur genome in two identical copies (Table S3). More recently, core components of the T9SS secretion system were found on the plasmid of *Cardinium c*BtQ1 (Santos-Garcia et al., 2014).

Originally described in *Flavobacterium johnsoniae*, the T9SS is unique among the phylum *Bacteroidetes* having important role in secretion of proteins involved both in gliding motility and pathogenicity (McBride & Nakane, 2015; Sato et al., 2010). The presence of the Gld homologs in *c*Cpun as well as *A. asiaticus* supports an ancestral origin of the T9SS machinery which was subsequently lost from *c*Eper1 and the nematode clade (*c*HgTN10 and *c*Ppe). The functional role of the T9SS components in *Cardinium* is unknown. The gene set identified as present in the clade is small compared to that known for active T9SSs (which may have more than 18 components). The low number of genes identified may either reflect co-option of other (unidentified) genes into the secretion process, or a function outside of secretion. However, it is tempting to speculate that the T9SS machinery in Amoebophilaceae has progressively been replaced by the AFP-like protein secretion system. This hypothesis is supported by the complete absence of Gld homologs in both *c*Eper1 and the nematode strains, which suggests that the T9SS is dispensable and likely undergoing gradual loss due to genome reduction processes (Toft & Andersson, 2010).

### The *c*Cpun genome contains an expansion of the DUF1703 gene family

Expansion and contraction of gene families in microbial genomes constitute a major source of both genetic and functional novelty, contributing to their adaptation to changing environments (Bratlie et al., 2010). Despite a tendency for evolution to eliminate redundancy and streamline genomes, endosymbiotic bacteria and intracellular pathogens often contain multi-gene families. Interestingly, the majority of the expanded gene families in these host-associated microbes encode putative effector proteins enriched in eukaryotic domains including ANK, LRR, and TPR repeats, F-box and U-box domains (Domman et al., 2014; Wu et al., 2004; Siozios et al., 2013; Schmitz-Esser et al., 2010).

**Figure 4:**
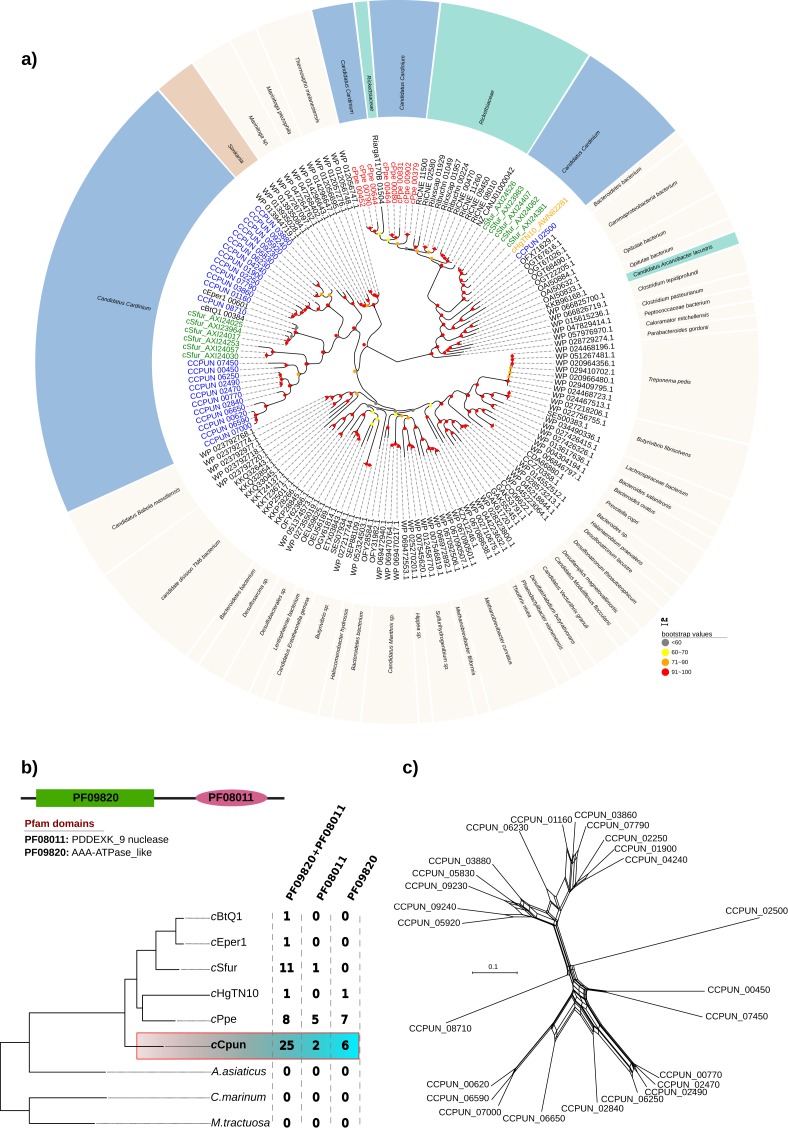
DUF1703 expansion in *c*Cpun genome. (A) Phylogenetic analysis of the *c*Cpun DUF1703 gene family. The unrooted phylogeny was inferred using maximum likelihood from the amino acid sequences of 156 DUF1703 homologs using IQ-TREE v1.6.6 (method: automated best model selection). *Cardinium*, *Simkania,* and *Rickettsia* homologs are shaded in blue, red, and green respectively. (B) The unique expansion of *c*Cpun DUF1703 gene family within the Amoebophilaceae. (C) Phylogenetic network showing the reticulated evolution of the *c*Cpun DUF1703 paralogs.

Inspection of the *c*Cpun genome revealed the presence of an expansion of hypothetical proteins related to the DUF1703 protein family (Knizewski et al., 2007) not previously observed in other *Cardinium* genomes, or other heritable microbes. A total of 25 gene paralogs coding for hypothetical proteins of this family were identified (Fig. 4). The DUF1703 family contains a group of modular proteins consisting of an N-terminal AAA-ATPase like domain (Pfam ID: PF09820) and a C-terminal PDDEXK_9 nuclease domain (Pfam ID: PF08011). In addition to the 25 paralogs, six genes were found to contain only the AAA-ATPase like domain whilst two genes contained only the nuclease domain (Fig. 4B). All partial genes were detected near the borders of the *c*Cpun scaffolds and may be artefactually truncated. Our estimate of gene family size is thus conservative.

The members of the DUF1703 gene family display in *c*Cpun are diverse, as attested by an average amino acid identity of just 39% amongst members (Fig. S5). This extensive divergence of paralogs suggests that the expansion of this gene family is not recent. Moreover, the pairwise comparison suggest at least three main expansion waves (Fig. S5). Phylogenetic analysis indicates that all but one of the *Cardinium c*Cpun DUF1703 carrying protein sequences form a single cluster closely related to those found in *Simkania*, an intracellular bacterium member of Chlamidiales known to be associated with protozoa (Fig. 4A). Notably, two of the simkania’s paralogs are encoded on its pSn plasmid, suggesting possible roots for horizontal dissemination of the DUF1703 genes. The exception is the gene CCPUN_02500, which forms a distinct group with homologs identified in *Cardinium* strain *c*Sfur and the only intact DUF1703 carrying homolog in cHgTN10, and which is closely related to homologs found in *Rickettsia* and metagenomically-recovered sequences belonging to uncultured members of the Bacteroidetes and Gammaproteobacteria (Anantharaman et al., 2016).

Although larger, the expansion of the DUF1703 gene family is not unique to the *c*Cpun genome. Amongst the most recently sequenced *Cardinium* genomes (*c*Sfur and *c*Ppe) we identified smaller expansions of the DUF1703 family (Figs. 4A and 4B). In contrast, the genomes of *c*Eper1, *c*BtQ1, and *c*HgTN10 contain only a single gene homolog whilst no homologs were detected in *A. asiaticus* or free-living relatives (Fig. 4B). Reconstruction of the phylogenetic relationships between the homologs clearly show that members from the same organism group together suggesting that independent expansions took place after divergence from the common ancestor. Surprisingly, the eight paralogs identified in *c*Ppe genome are more closely related to their *Rickettsia* counterparts than the rest of the *Cardinium* homologs, indicating possible independent acquisition. Our results suggest that the DUF1703 genes have probably originated in *Cardinium* after they diverged from *A. asiaticus*, presumably by horizontal gene transfer (HGT) with later expansion in the lineage leading to *c*Cpun, *c*Sfur, and *c*Ppe.

Phylogenetic network analyses revealed several reticulation events within the DUF1703 gene family in *c*Cpun indicating frequent recombination among gene family members (Fig. 4C). We further investigated the extent of recombination using different methods implemented in RDP4 software (Martin et al., 2015). Due to the limited sequence similarity between the members of the DUF1703 family we restricted our analyses to group of sequences sharing at least 65–70% nucleotide similarities since misalignment artefacts can confound the identification of true recombination signals. We detected evidence of intragenic recombination in all examined groups with multiple methods (Table S4) suggesting that DUF1703 paralogs in *c*Cpun readily recombine. Despite the extensive recombination, no apparent homogenization between the members of this gene family is observed as suggested by the limited sequence similarity and the absence of monophyletic clustering of *c*Cpun paralogs. Overall, our results point to a HGT scenario for the origin of *Cardinium* DUF1703 gene family with subsequent expansion in the *c*Cpun genome, and variation produced both by mutation and recombination.

**Figure 5:**
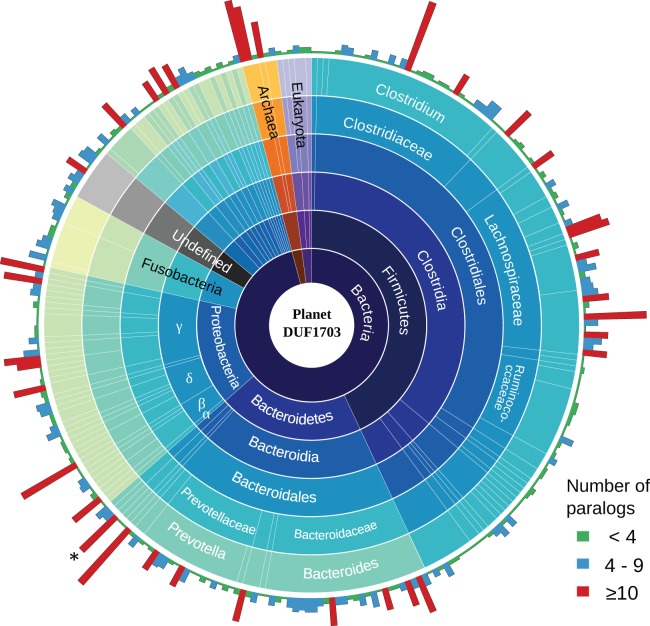
Planet DUF1703. Abundance and taxonomic distribution of DUF1703 proteins in PFAM database. *: cCpun genome. The graph was constructed using Circos v0.69 (Krzywinski et al., 2009).

To gain a better insight into the role of DUF1703 proteins we sought to investigate the distribution and abundance of proteins containing the AAA-ATPase and PDDEXK_9 domains in other prokaryotes and eukaryotes. We searched the Pfam database for protein sequences containing the two domains and exhibited similar architecture with *Cardinium* homologs. In most cases, DUF1703 containing genes occurred in low copy number per genome. Most species carried fewer than four copies whilst only 9.8% of the species contained 10 copies or more (Fig. 5), ranking *c*Cpun among the species with the largest number of DUF1703 paralogs. Species with higher abundance of DUF1703 paralogs are scattered across the prokaryotic taxonomy suggesting that DUF1703 protein expansion has occurred on multiple occasions within bacteria.

The reason for the expansion of the DUF1703 gene family in *c*Cpun and its putative functional role is yet unknown. It is notable that DUF1703 genes have been also identified in the *Rickettsia* endosymbiont infecting biting midges (Pilgrim et al., 2017). Mirroring the pattern for midge *Cardinium*, the midge *Rickettsia* genome also contains multiple DUF1703 paralogs compared to other *Rickettsia* species with evidence of intragenic recombination (*p* < 0.001, PHI test, 1,000 permutations). However, *Cardinium c*Cpun and *Rickettsia* DUF1703 carrying genes are phylogenetically unrelated (Fig. 4A) suggesting independent evolutionary histories, and independent expansion of this gene family in the two groups of midge symbionts. These data suggest this gene family may have a particular function in symbiosis with midges.

The biological role of the DUF1703 is still unclear. A recent transcriptomic study of the *Cardinium* strain *c*Eper1 in its host *E. suzannae* showed that its only DUF1703 gene homolog is moderately transcribed in both sexes (Mann et al., 2017). Notably, a putative signal peptide cleavage site was predicted for 10 out of 25 DUF1703 paralogs in *c*Cpun (Table S5) suggesting that they are potentially secreted, acting against DNA/RNA outside of the symbiont. Surprisingly, no signal peptides were detected in any of the paralogs identified in *c*Sfur and *c*Ppe (data not shown). It is noteworthy that an intact DUF1703 homolog of bacterial origin has been previously reported as component of the Maternal-Effect Dominant Embryonic Arrest (“MEDEA”) factor, a selfish genetic element reported in *Tribolium castaneum* (Lorenzen et al., 2008). PD-(D/E)XK nucleases constitute a large and functionally diverse superfamily of proteins which includes among others restriction endonucleases, Holliday junction resolvases, transposases, and DNA repair enzymes (Steczkiewicz et al., 2012). Recently, dual PD-(D/E)XK nuclease domains have been identified in a wide range of toxins from diverse intracellular bacteria (Gillespie et al., 2018; Lindsey et al., 2018). More interestingly, some of these domains have been directly linked with the induction of reproductive parasitism in the form of CI in *Wolbachia* (Beckmann, Ronau & Hochstrasser, 2017). Structural comparison of the PD-(D/E)XK core nuclease site from *Cardinium* and *Rickettsia* DUF1703 homologs and that of the CI-like toxins show considerable differences, especially in the sequence between the catalytic residues (Asp, Glu, and Lys) (Fig. S6). In addition, the AAA-ATPase domain associated with the DUF1703 nuclease is not found in the CI-like toxins of *Wolbachia* and related proteins (Gillespie et al., 2018) which might suggest these proteins have different functions. The biological role of *Cardinium* DUF1703 proteins remains to be determined.

### Putative horizontal gene transfers as a source of genes in the *c*Cpun genome

Horizontal gene transfer has been previously reported as the source of several genes in *A. asiaticus*, *c*Eper1, and *c*BtQ1 (Penz et al., 2012; Santos-Garcia et al., 2014; Schmitz-Esser et al., 2010). Many of the HGT genes were found to be shared with members of the Alphaproteobacteria that have an intracellular lifestyle, especially species within the *Rickettsiales* order, consistent with HGT within the shared environment of the cell.

In accordance with previous observations of symbiont genomes, our results indicate that HGT has likely shaped the accessory genomes of *c*Cpun (Table 2). The majority of the accessory genes of *c*Cpun for which homologs could be assigned in the database are more similar to corresponding genes of bacterial species outside *Bacteroidetes,* with a bias to genes within the Proteobacteria having closest sequence similarity (Table 2; Fig. S7). For *c*Cpun-specific genes, closest sequence matches lay within bacterial species known to be associated with other arthropods including *Rickettsia* and *Wolbachia*, as well as the amoeba-associated bacteria *Candidatus* Paracaedibacter acanthamoebae and *Candidatus* Jidaibacter acanthamoeba (Table 2). Four of these genes clustered with gene sequences from torix group *Rickettsia*, which are also found in midges. Three of these genes encode putative transposases, and one is a hypothetical protein that in other *Rickettsia* is located on a plasmid hypothesized to be important in determining the host-symbiont interaction (Gillespie et al., 2015).

**Table 2:** Example of *c*Cpun genes likely originated from HGTs.

Gene id	Length (AA)	Annotation	Taxonomy of the Best BLAST hit, (GenBank accession)	*E*-value	AA identity (%)
CCPUN_00040	308	Hypothetical protein, putative transposase	*Rickettsia* endosymbiont of *Culicoides newsteadi*, (WP_094649760)	2E-128	64
CCPUN_00530	328	Hypothetical protein, putative transposase	*Rickettsia* endosymbiont of *Culicoides newsteadi*, (WP_094649760)	3E-124	62
CCPUN_01090	346	Hypothetical protein, putative transposase	Rickettsiales bacterium, (PCJ29205)	6E-133	58
CCPUN_02050	379	Hypothetical protein, putative transposase	Rickettsiales bacterium, (PCJ24349)	5E-55	44
CCPUN_04150	328	Hypothetical protein, putative transposase	*Rickettsia* endosymbiont of *Culicoides newsteadi*, (WP_094649760)	9E-125	59
CCPUN_04430	297	Hypothetical protein, putative transposase	Rickettsiales bacterium, (PCJ25778)	9E-136	65
CCPUN_01120	218	Carbonic anhydrase	*Lysobacter sp*. Root494, (WP_056131435)	2E-95	59
CCPUN_03570	551	DNA repair protein RecN	Rickettsiales bacterium, (PCJ29272)	2E-175	48
CCPUN_03900	258	Hypothetical protein, putative transposase	*Candidatus* Paracaedibacter acanthamoebae, (WP_038464592)	3E-114	67
CCPUN_06490	469	Arginine/agmatine antiporter	Gammaproteobacteria bacterium 39-13, (OJV90723)	4E-112	43
CCPUN_07910	266	Chromosome-partitioning protein Spo0J	*Candidatus* Phycorickettsia trachydisci, (WP_106874767)	9E-101	57
CCPUN_07920	327	Sporulation initiation inhibitor protein Soj	*Candidatus* Phycorickettsia trachydisci, (WP_106874768)	5E-135	62
CCPUN_08840	436	Folylpolyglutamate synthase	Wolbachia pipientis, (WP_010963010)	0E+00	76
CCPUN_08910	340	Hypothetical protein	*Rickettsia felis*, (WP_039595314)	2E-155	73
CCPUN_03830	426	Hypothetical protein	*Aedes aegypti*, (XP_001656120)	2E-60	39
CCPUN_08280	1,360	Hypothetical protein	Aedes albopictus, (KXJ68548)	5E-72	27

Among the putatively horizontally exchanged gene set were ORFs encoding a carbonic anydrase (CA), an amino acid permease, and a putative chromosome-partitioning protein. Finally, two *c*Cpun-specific genes encoding hypothetical proteins had their closest homologs within *Aedes* mosquitoes (Table 2; Fig. S7). Notably, the two proteins also have partial similarities with a large ankyrin repeat containing protein (Aasi_1610) previously identified in *A. asiaticus* (Schmitz-Esser et al., 2010). Although both *c*Cpun proteins had their ten top hits assigned to *Aedes* sequences, the partial similarities to *A. asiaticus* suggest that they might be fragments of an Aasi_1610 distant homolog. Note, the number of these genes derived from HGT may be even higher since the majority of the accessory genes did not have any significant matches on the GenBank database, and many of these likely represent HGT events from as yet uncharacterized genomes.

The presence of CAs gene is interesting. Among the Amoebophilaceae, CA homologs were detected only in *c*Cpun, *c*Sfur, and *c*HgTN10 and not in other *Cardinium* strains nor *A. asiaticus*, Notably, the three *Cardinium* homologs do not form a monophyletic group, with *c*HgTN10 and *c*Sfur homologs being clustered together and more closely associated with a putative CA previously identified in the *Rickettsia* endosymbiont previously found in biting midges (Pilgrim et al., 2017) (Fig. S8). Our results suggest that the *Cardinium* CA homologs have independent evolutionary histories and probably originated from independent horizontal transfer events into the three genomes.

The function of these CAs is not clear. CAs are ancient and ubiquitous multi-class zinc-containing metalloenzymes that catalyze the interconversion of CO_2_ to bicarbonate (Smith & Ferry, 2000; Smith et al., 1999) and are involved in a variety of biochemical processes including respiration and pH homoeostasis (Gai et al., 2014). Studies have shown that CAs are essential for microbial growth in free living bacteria under ambient air with low levels of CO_2_ (Mitsuhashi et al., 2003; Merlin et al., 2003; Kusian, Sültemeyer & Bowien, 2002). However, whilst CAs are common in many bacterial groups, they are less commonly observed in the genomes of obligate intracellular bacteria (Ueda, Nishida & Beppu, 2012). Studies suggest that intracellular pathogens may rely on CAs for virulence and survival within the host cell (Valdivia & Falkow, 1997), possibly through regulating the phagosome pH during the infection (Nishimori et al., 2014). An intriguing hypothesis is whether CAs might actually play a role in the survival of *Cardinium* outside of the host in comparable way to the role of CAs in free living bacteria, and thus facilitating its horizontal transmission. Interestingly, the plant-mediated horizontal transmission of *Cardinium* bacteria between phloem sap-feeding insects has been previously reported, supporting such a scenario (Gonella et al., 2015).

*c*Cpun lacks a biotin or other B-vitamin biosynthetic pathways, indicating it is unlikely to act as a source of these vitamins to its haematophagous host. Indeed, putative homologs of the complete biotin transport system (BioY: CCPUN_01590, BioM: CCPUN_08370, and BioN: CCPUN_08380) were detected, suggesting that *c*Cpun may depend on external provision of biotin from the host. The presence of a complete biotin transporter gene set contrasts with other *Cardinium* genomes, which lack these transporters, but may carry complete operons for the synthesis of biotin, lipoeta and pyridoxal 5’-phosphate (vitamin B6) (Penz et al., 2012). Exception is the recently sequenced strain *c*Sfur which encode for both a biotin transport system and a complete operon for biotin synthesis (Zeng et al., 2018).

## Conclusions

In the present study, we expanded the current genomic information from *Cardinium* lineages by presenting a new *Cardinium* draft genome belonging to the divergent and poorly studied group C. Phylogenomic comparison clearly nests the B group nematode-associated *Cardinium* symbionts within the clade A and C symbionts derived from insect strains, indicating that inference previously made on the basis of two gene sequences can now be regarded as supported robustly. The lack of monophyly of strains of *Cardinium* symbiotic with arthropods resembles the pattern for *Wolbachia*, where nematode *Wolbachia* strains are nested within a diverse set of arthropod *Wolbachia* strains (Gerth et al., 2014). Heritable microbes occasionally switching between distant host phyla may be more common than previously considered, with the pattern observed in *Wolbachia* (nematode and arthropod infections), torix *Rickettsia* (leech and arthropod lineages) and here in *Cardinium*.

Comparative genomics also provides some insight into whether the three *Cardinium* clades consist different species. The assignment of systematic names in symbiotic bacteria has been a controversial field, owing to the intimate association with their hosts and their ability to exchange genetic material. Nakamura et al. (2009) had previously proposed the use of the single species name “*Candidatus Cardinium hertigii*” to describe the three *Cardinium* clades (A, B, C) based on morphological similarities and comparable substitutions in the 16S rRNA gene with other symbiotic bacteria. The paucity of *Cardinium* genomic data and the complete absence of phenotypic information on all but clade-A suggest that is still early to apply an accurate systematic framework. However, the extensive genomic diversity between *Cardinium* clades suggest that *Cardinium* clades may be best described as separate species. Future genomic and phenotypic data will allow us to revise the taxonomy within *Cardinium* lineage.

The presence of *Rickettsia* alongside *Cardinium* in midges presents an opportunity to examine whether the genomes show any convergent properties and if HGT has occurred. Comparison of the gene content of the *c*Cpun *Cardinium* strain with the RiCNE *Rickettsia* symbiont of *Culicoides newsteadi* revealed some similarities. Expansion of the DUF1703 gene family and presence of a carbonic anhydrase gene were notable. However, neither case reflects HGT in the intracellular environment of midges, with the same pattern being independently derived. This separate derivation indicates the possession of these genes may be convergent properties, biologically related to symbiotic life in biting midge hosts, rather than HGT within a shared environment.

Finally, our data indicate that the *Cardinium* symbiont in biting midges is unlikely to serve as a source of B vitamins to its haematophagous host. Contrary to the *c*Eper1 genome, a biotin synthesis system was not observed in the *c*Cpun genome, and indeed the presence of a biotin transporter system indicates the symbiont may in fact be an importer of biotin, and thus a B vitamin sink rather than source. This result perhaps reflects the mixed trophic relationship of biting midges, where larval phases are aquatic and detritivores, and the adult phase either haematophagous (female) or reliant only on sugar sources (males). It is likely that B vitamins are acquired heterotrophically in the larval phase in sufficient quantities such that selection for symbiont-mediated supplementation is low. Given that a major vector species, including *Culicoides imicola,* harbours *Cardinium* (Morag et al., 2012), future work should likely focus on their effects on vectorial capacity alongside the putative facilitation of *Cardinium*-midge interactions from the DUF1703 gene family and carbonic anhydrases.

## Supplemental Information

10.7717/peerj.6448/supp-1 Supplemental Information 1 Taxon annotated GC-coverage plot of the primary genome assembly of *Culicoides punctatus*.

10.7717/peerj.6448/supp-2 Supplemental Information 2 Circular representation of *c*Cpun genome using Circos v0.69 (Krzywinski *et al.* 2009). To enhance visualization the scaffolds were size sorted and concatenated into a pseudomolecule. The alternating grey and white strips highlight the scaffold borders. Inwards, the first, second and third circle are colour coded according to COG functional categories and represent a) the complete *c*Cpun protein coding genes, b) the core genes between the four *Amoebophilaceae* genomes presented in Figure 1A, and c) the *c*Cpun unique genes. In the fourth circle we show the genomic location of the genes coding for the Afp-like (magenta) and type IX (red) secretion systems as well as the DUF1703 gene paralogs. Finally, the two line plots represent genome coverage and GC% content across *c*Cpun genome (1kb sliding window) respectively. An orange line indicates the mean coverage (90X) of the draft assembly.

10.7717/peerj.6448/supp-3 Supplemental Information 3 *c*Cpun is a near complete genome. BUSCO completeness assessment results for *c*Cpun draft genome in comparison to the other *Cardinium* genomes and *A. asiaticus*. The Results are based on the presence or absence of 148 single-copy universal bacterial markers.

10.7717/peerj.6448/supp-4 Supplemental Information 4 Repeat-content comparison across the seven Amoebophilaceae genomes. Mummer self-plots representing sequence repeat density in the seven *Amoebaphilaceae* genomes. Each dot represent a repeat (red=direct) and (blue=inverted) of at least 200bp and 95% similarity.

10.7717/peerj.6448/supp-5 Supplemental Information 5 Pairwise similarity (lower right) and identity (upper left) matrix of the 24 DUF1703 protein paralogs identified in the genome of *c*Cpun.

10.7717/peerj.6448/supp-6 Supplemental Information 6 Structural analysis of the DUF1703 nuclease core site. a) Multiple sequence alignment of the DUF1703 nuclease domain from *Cardinium* (blue outline) and *Rickettsia* (red outline) homologs described in Fig. 4. The core PD-(D/E)XK site is highlighted in yellow and the catalytic Asp, Glu and Lys residues are indicated with an asterisk. b) Residue conservation within the core PD-(D/E)XK site between the DUF1703 nuclease family depicted in (a) and the type II-IV CI-like toxins of *Wolbachia* and related proteins described in Gillespie et al. (2018). Sequence logos were prepared using the Skylign tool (Wheeler, Clements & Finn, 2014).

10.7717/peerj.6448/supp-7 Supplemental Information 7 Phylogenetic analysis of the putative HGT genes of *c*Cpun genome. Phylogenetic relationships of *c*Cpun HGTs with their closest homologs in the Genbank database was inferred using maximum likelihood with IQ-TREE v1.6.6 (method: automated best model selection). Branch support values are based on 1000 bootstrap replicates.

10.7717/peerj.6448/supp-8 Supplemental Information 8 *Cardinium* carbonic anhydrase homologs. Maximum likelihood phylogenetic placement of *c*Cpun, *c*Sfur and cHgTN10 carbonic anhydrase (CAs) protein sequences compared with their closest homologs in the Genbank database. Members from the four clades forming the beta-class of CAs are presented. The positions of the *Cardinium* homologs and the CA homolog identified in *Rickettsia* (RiCNE) endosymbionds in biting midges are indicated in purple and red respectively. Phylogenetic relationships were inferred using IQ-TREE v1.6.6 (method: automated best model selection).

10.7717/peerj.6448/supp-9 Supplemental Information 9 Functional annotation of *c*Cpun draft genome including Pfam domains and eggNOG results.

10.7717/peerj.6448/supp-10 Supplemental Information 10 *c*Cpun unique genes.

10.7717/peerj.6448/supp-11 Supplemental Information 11 Type IX secretion system (T9SS) components in *c*Cpun genome.

10.7717/peerj.6448/supp-12 Supplemental Information 12 Evidences of recombination between the cCpun DUF1703 paralogs as determined with RDP4 software.

10.7717/peerj.6448/supp-13 Supplemental Information 13 Signal peptide prediction in the 25 intact DUF1703 protein paralogs of cCpun using the SignalP 4.1 server.

10.7717/peerj.6448/supp-14 Supplemental Information 14 Alignment files used in this study.

## Additional Information and Declarations

### DNA Deposition

The following information was supplied regarding the deposition of DNA sequences: The raw reads and the *c*Cpun draft genome assembly have been submitted to the DDBJ/EMBL/GenBank database under the BioProject accession number PRJNA487198 (WGS project QWJI00000000).

### Data Availability

The following information was supplied regarding data availability: The supermatrix file used for the phylogenomic analysis (Fig. 1) and the alignment files used for the phylogenetic analyses of the DUF1703 and the Carbonic Anhydrase gene families (Fig. 3; Fig. S6, respectively) are provided as supplementary files. Both trimmed and untrimmed versions of the alignment files are provided.
