# Supplementary figures and images for: The draft genome of strain cCpun from biting midges confirms insect Cardinium are not a monophyletic group and reveals a novel gene family expansion in a symbiont
Source: PeerJ. 2019 Feb 21;7:e6448. doi: 10.7717/peerj.6448 (PMC6387759; doi:10.7717/peerj.6448)

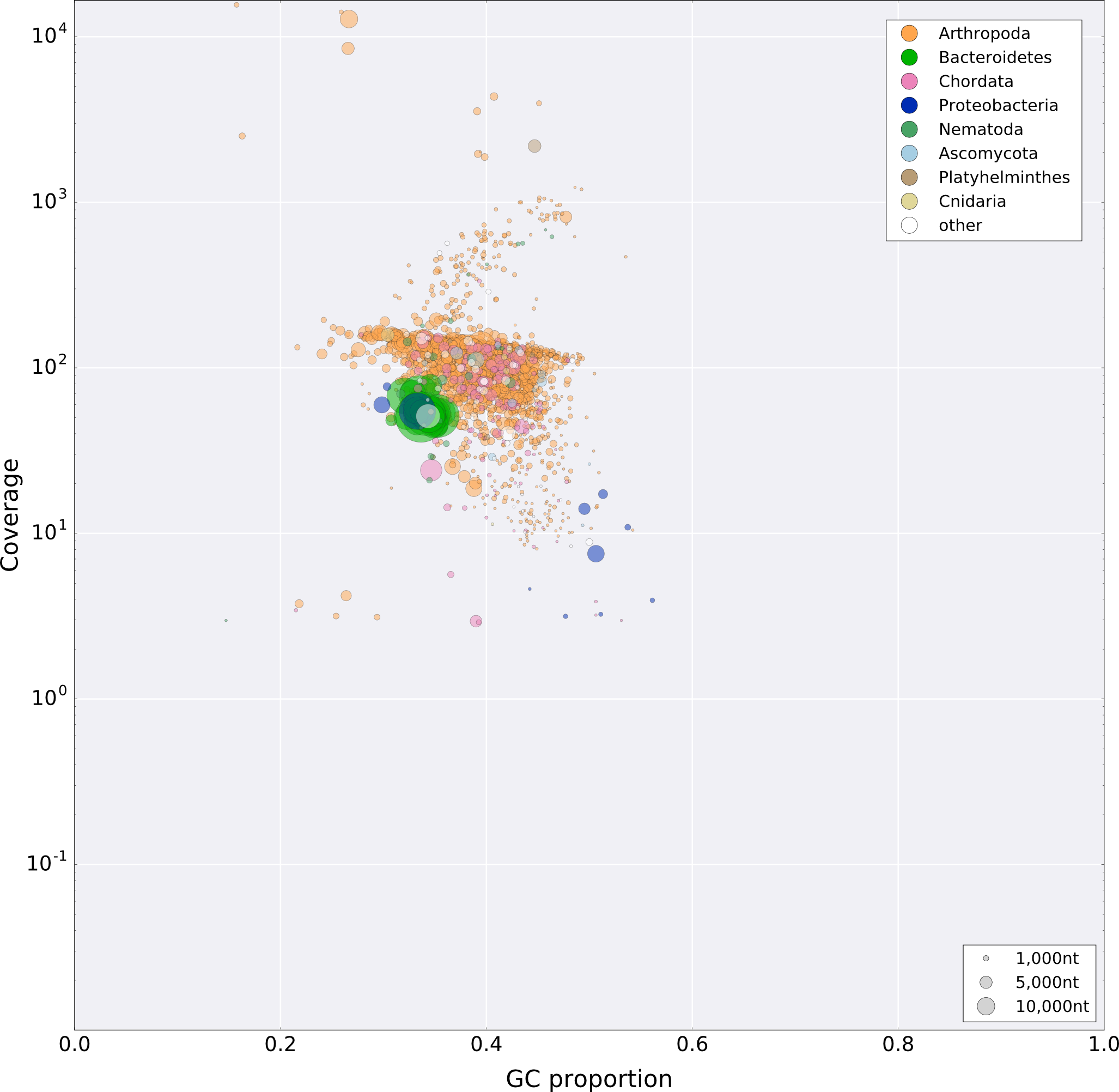

Supplement: Supplemental Information 1 [file peerj-07-6448-s001.png]

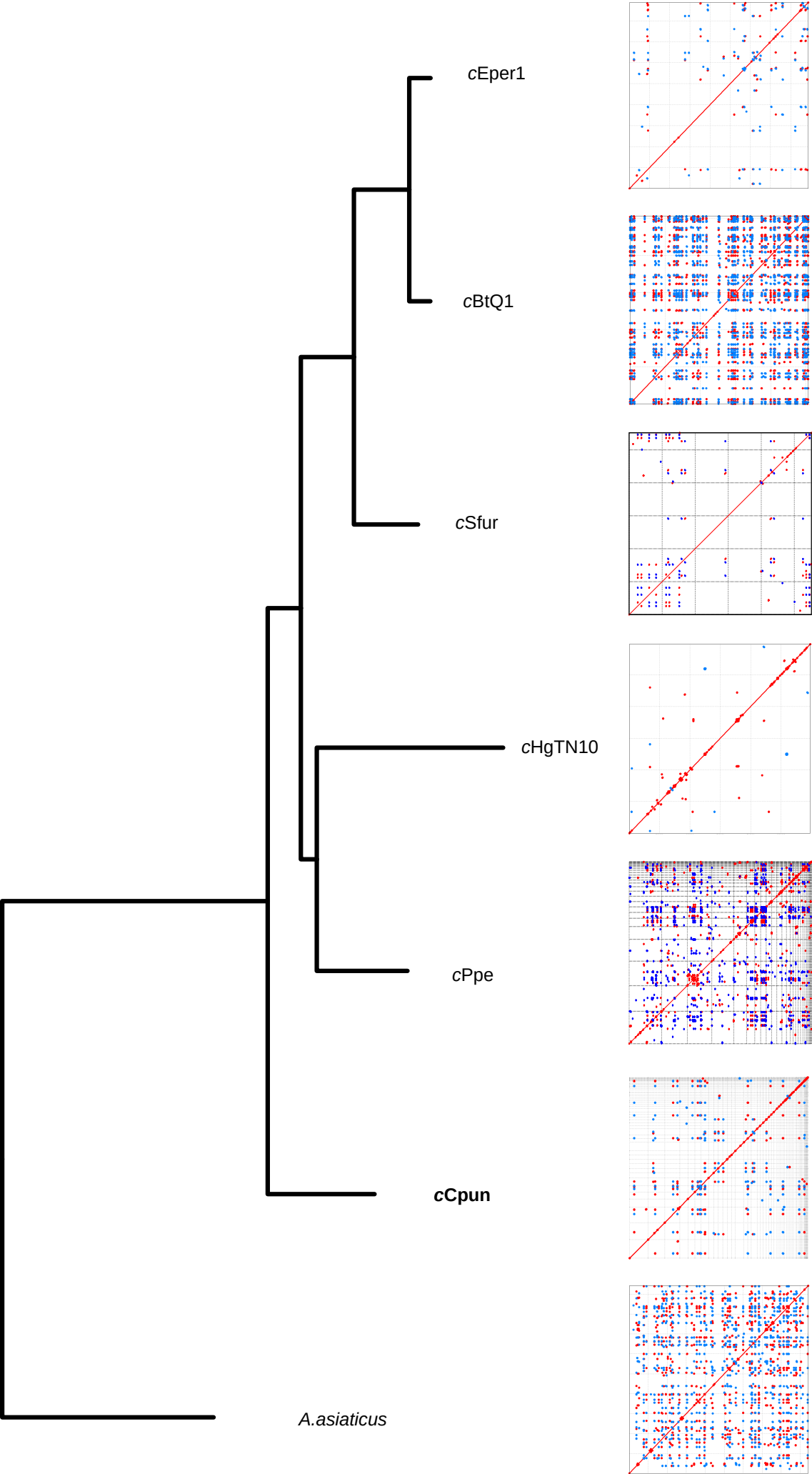

Supplement: Supplemental Information 4 — Mummer self-plots representing sequence repeat density in the seven Amoebaphilaceae genomes. Each dot represent a repeat (red=direct) and (blue=inverted) of at least 200bp and 95% similarity. [file peerj-07-6448-s004.pdf]

Color Key

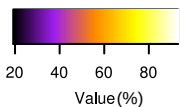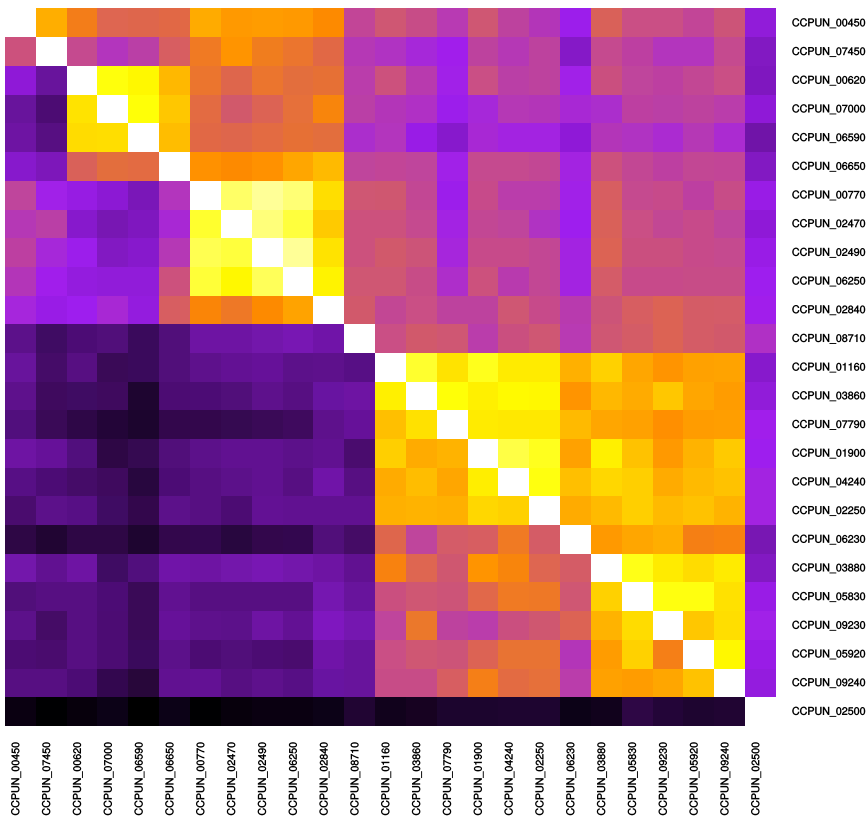

Supplement: Supplemental Information 5 [file peerj-07-6448-s005.pdf]

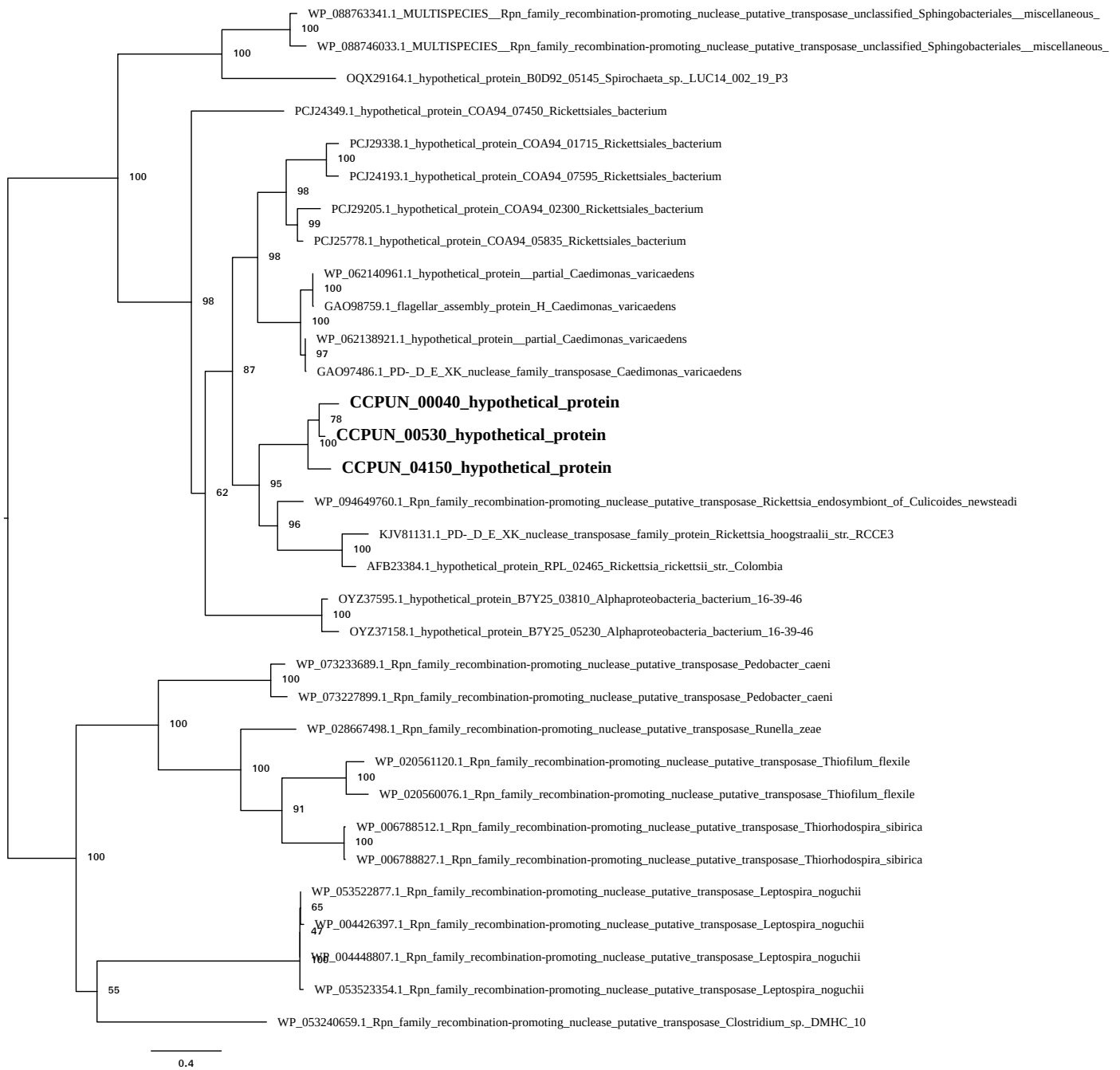

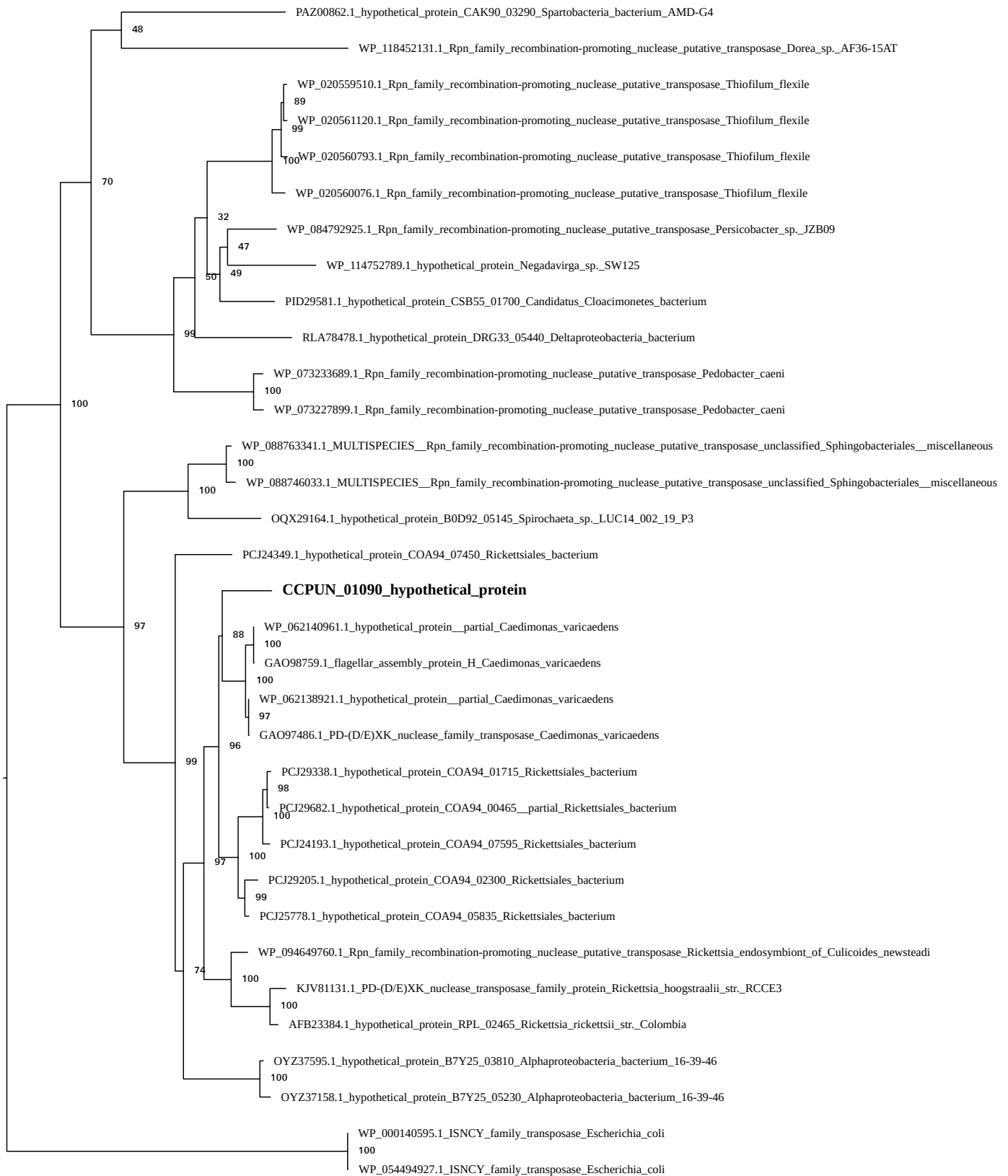

0.5

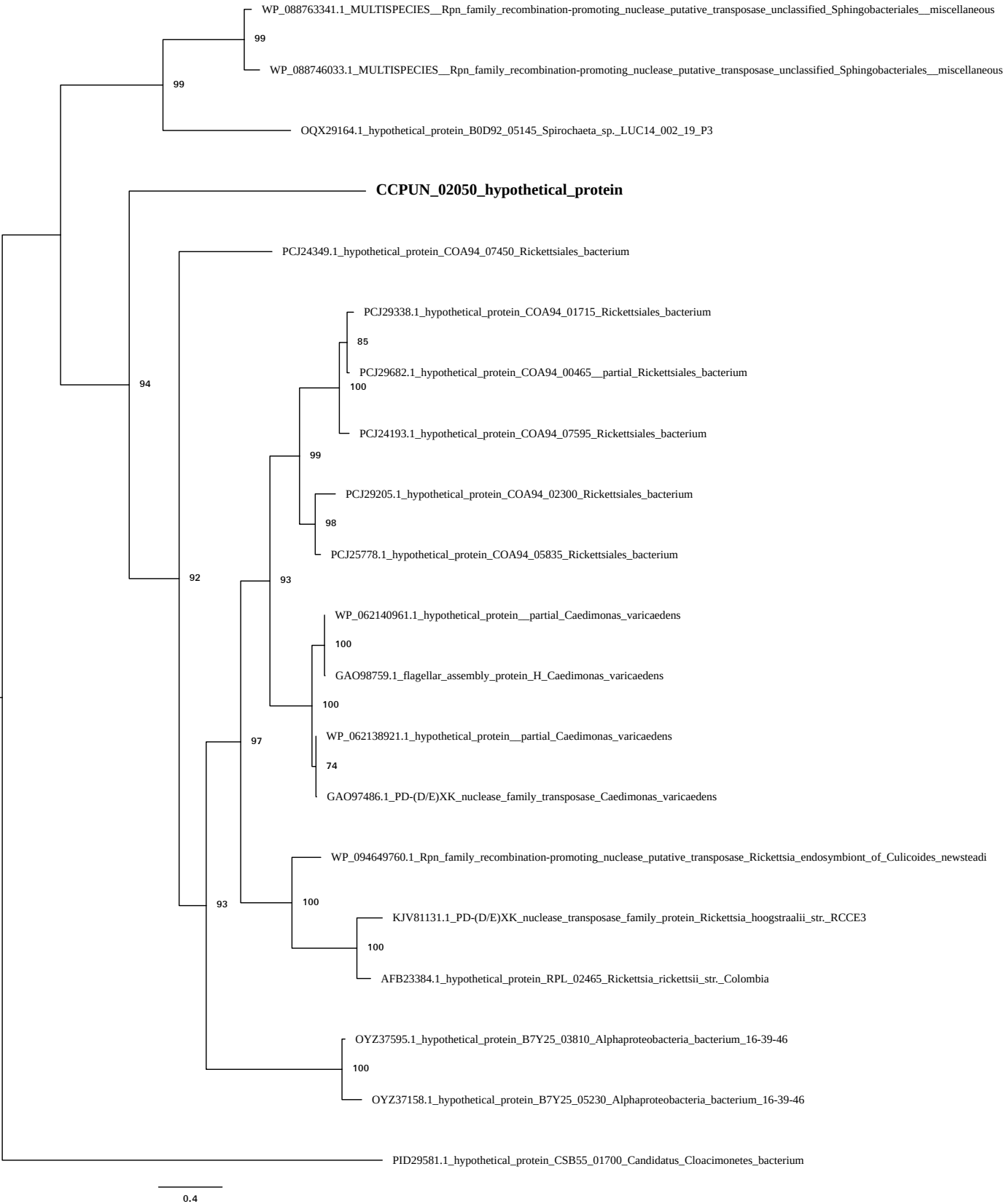

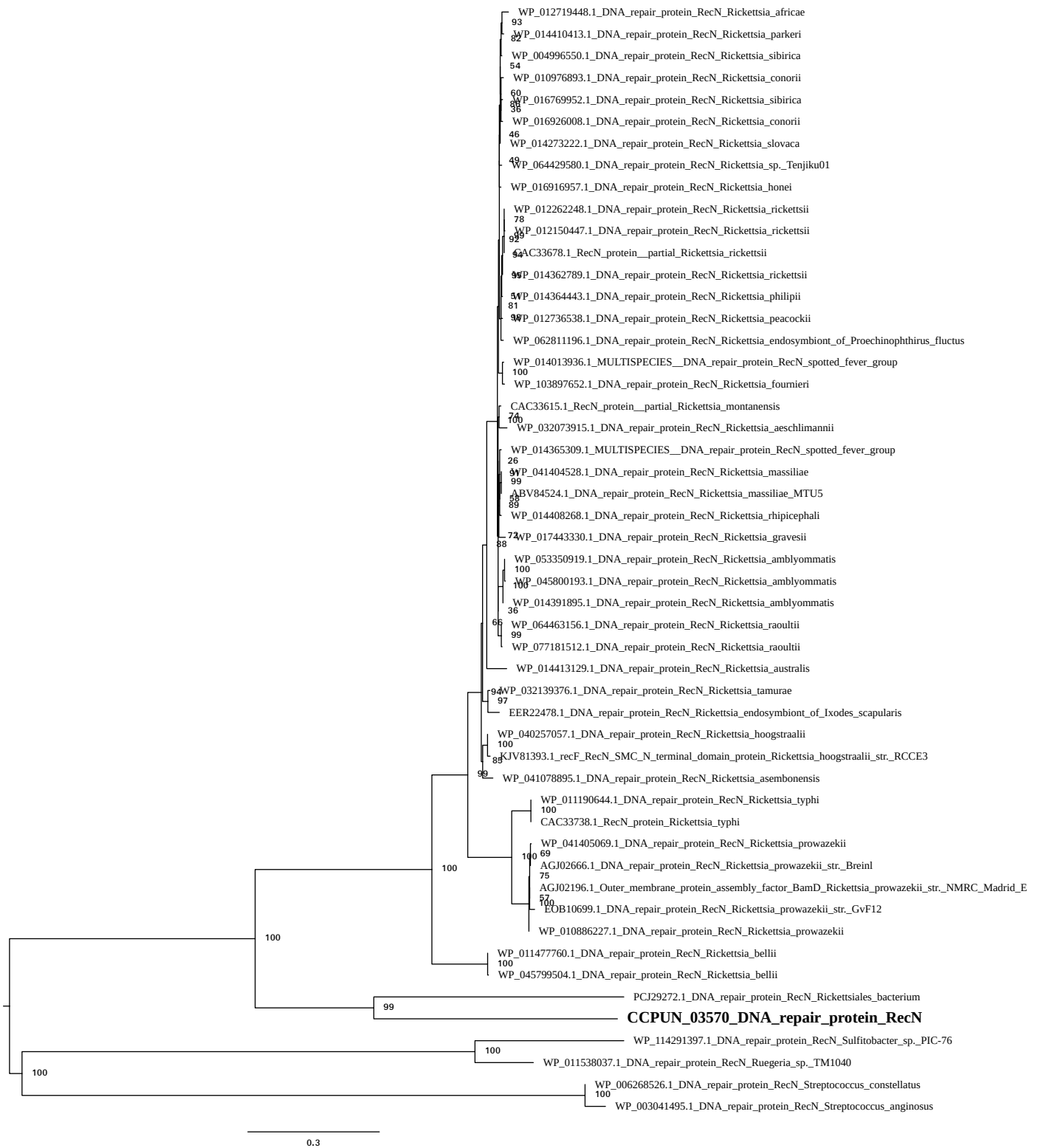

CCPUN\_03830

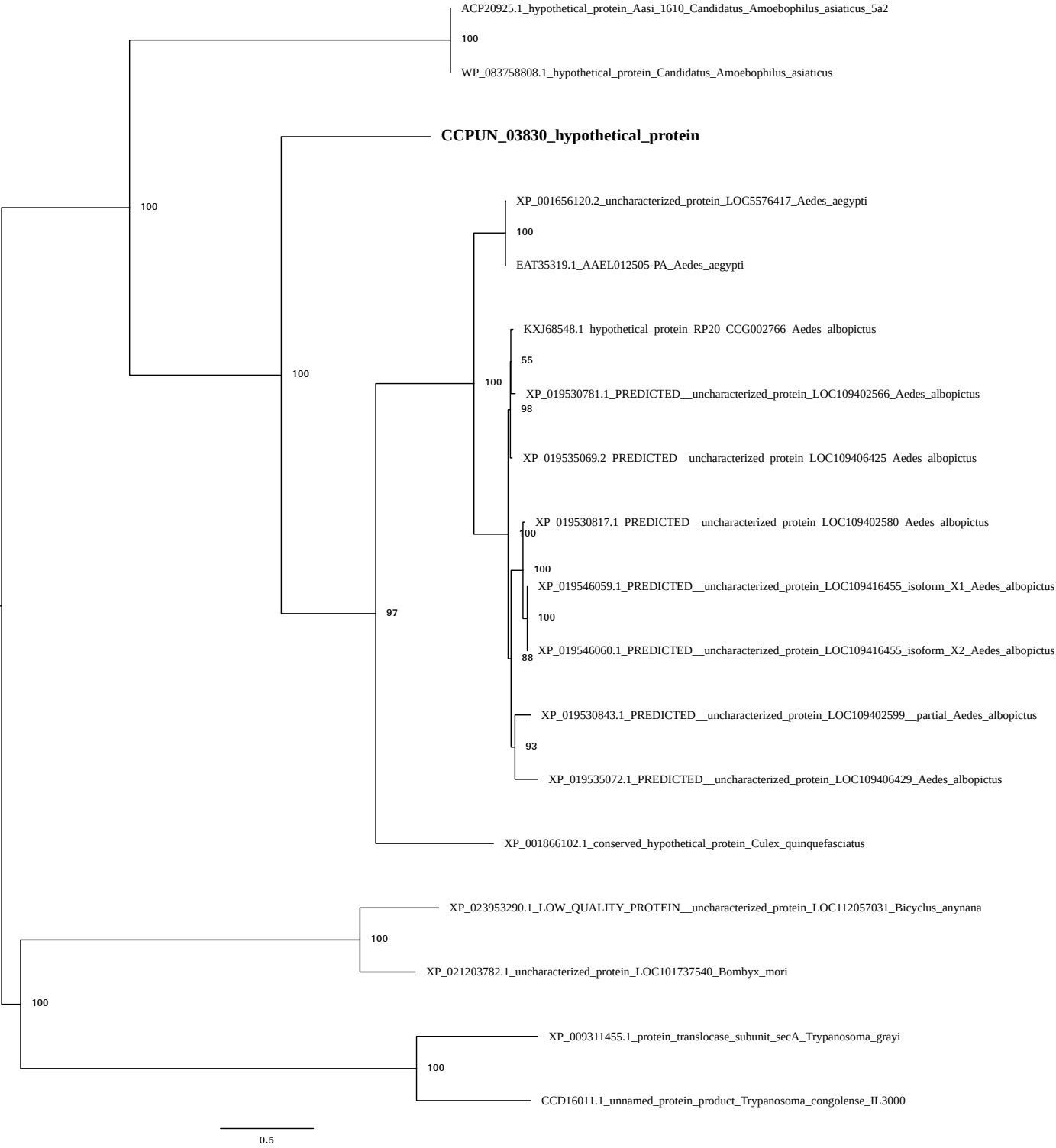

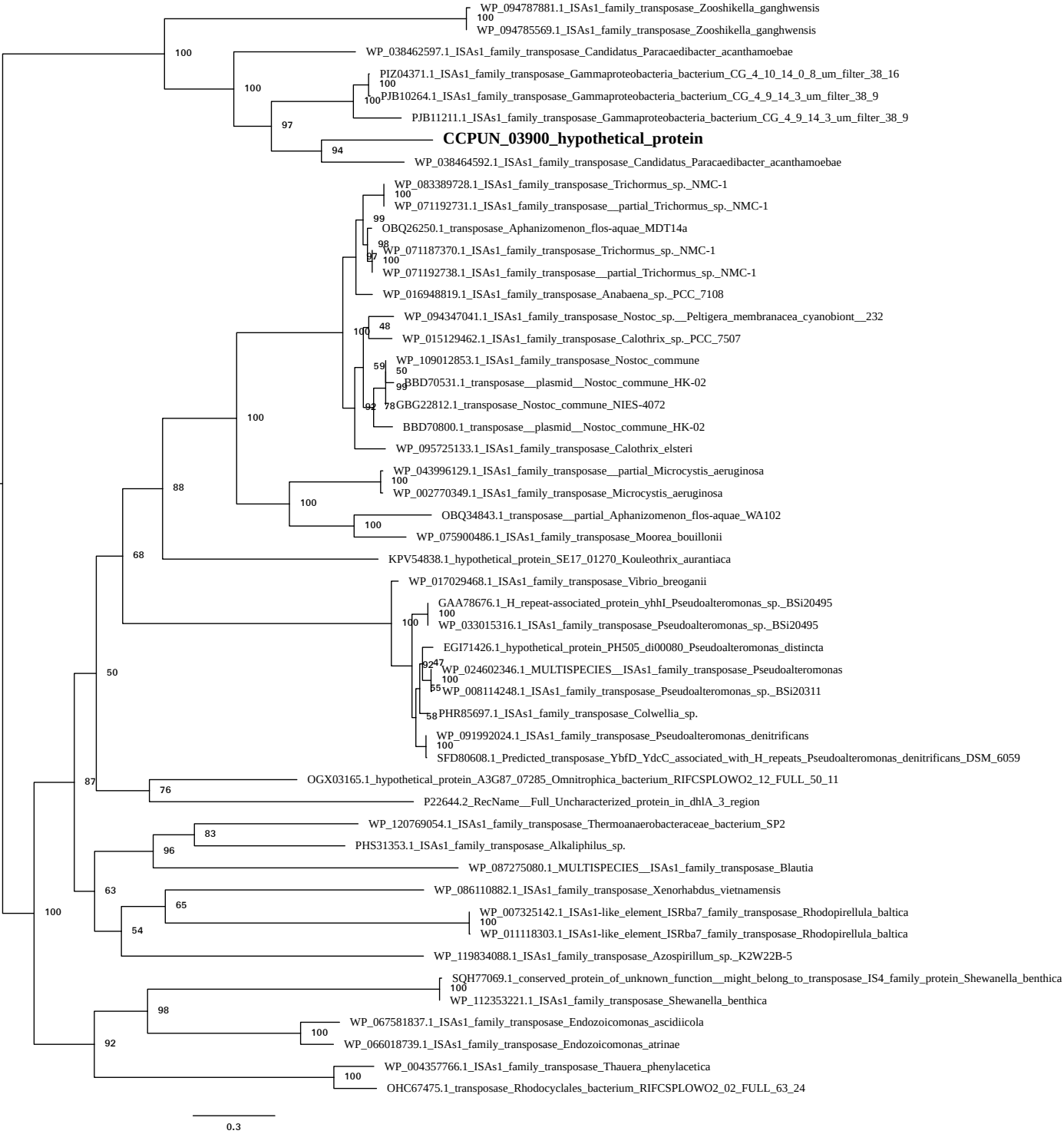

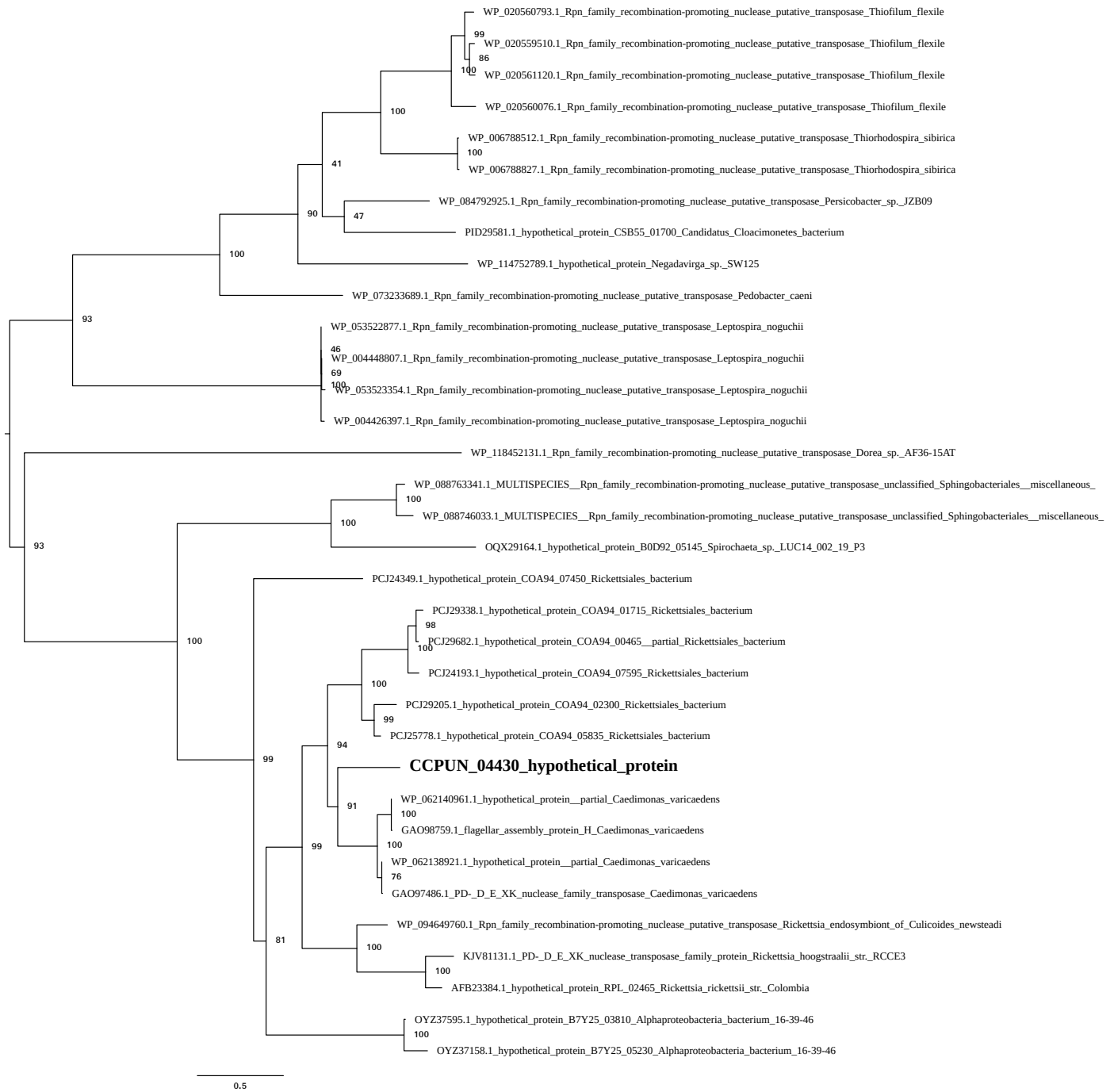

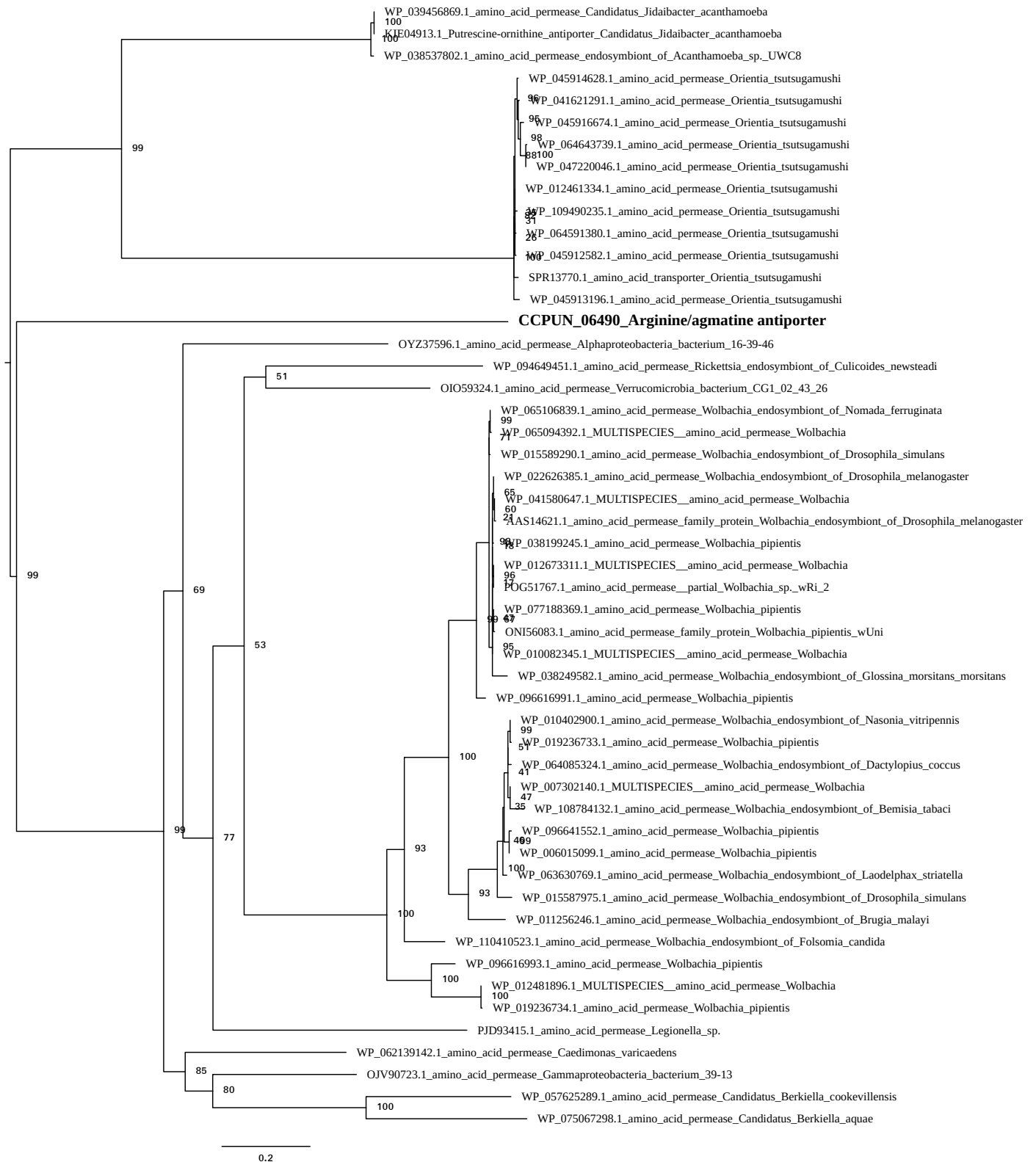

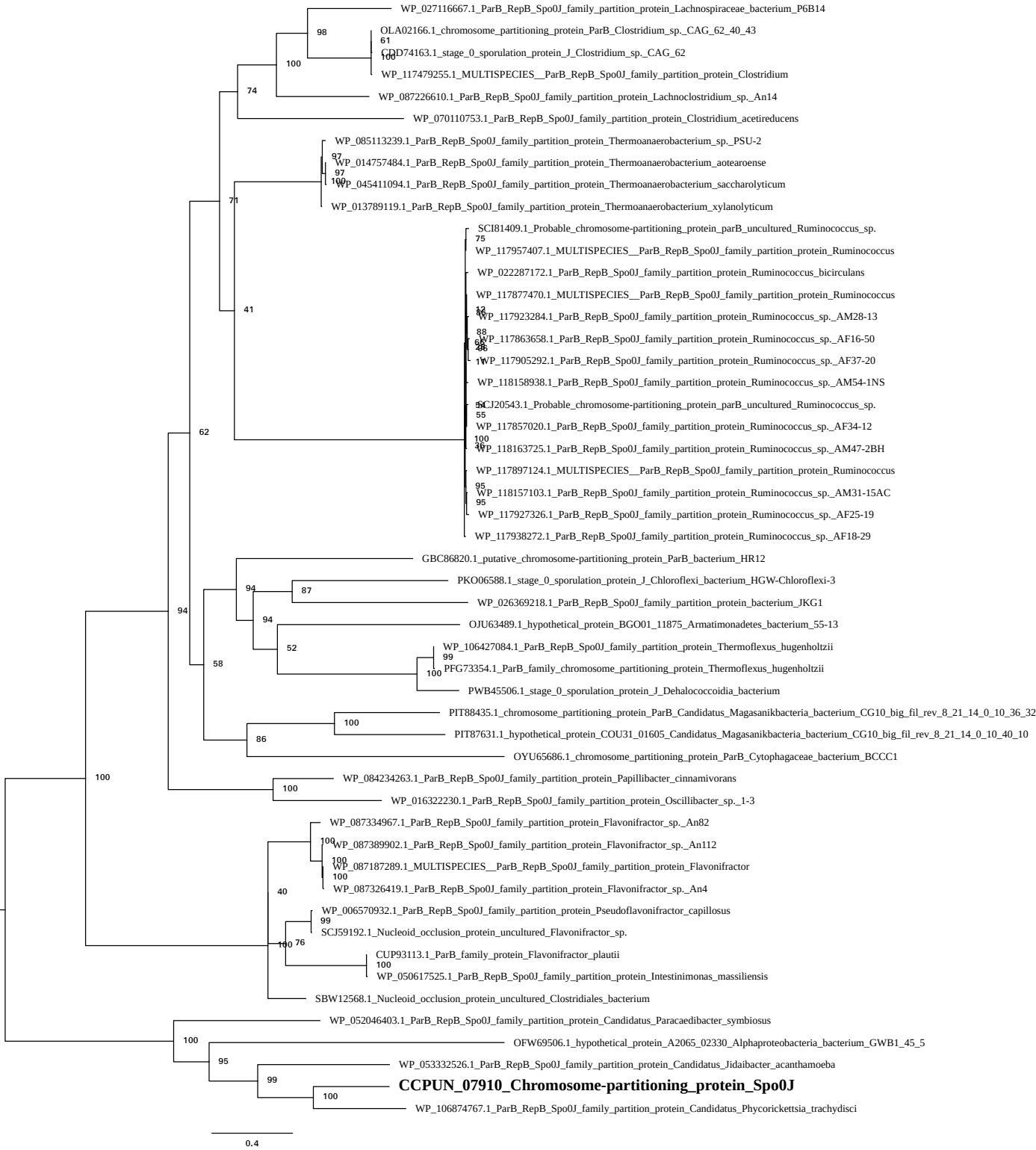

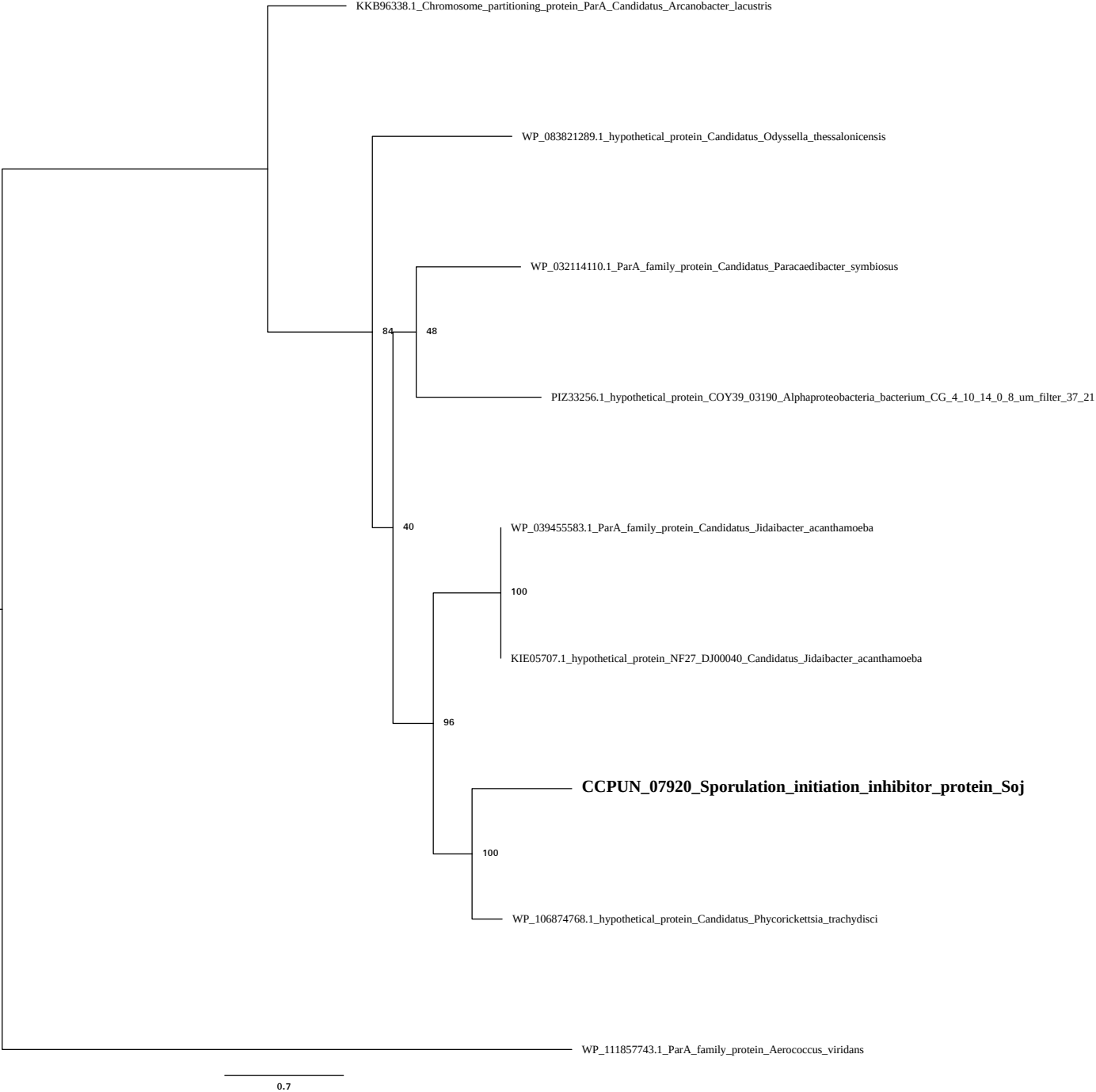

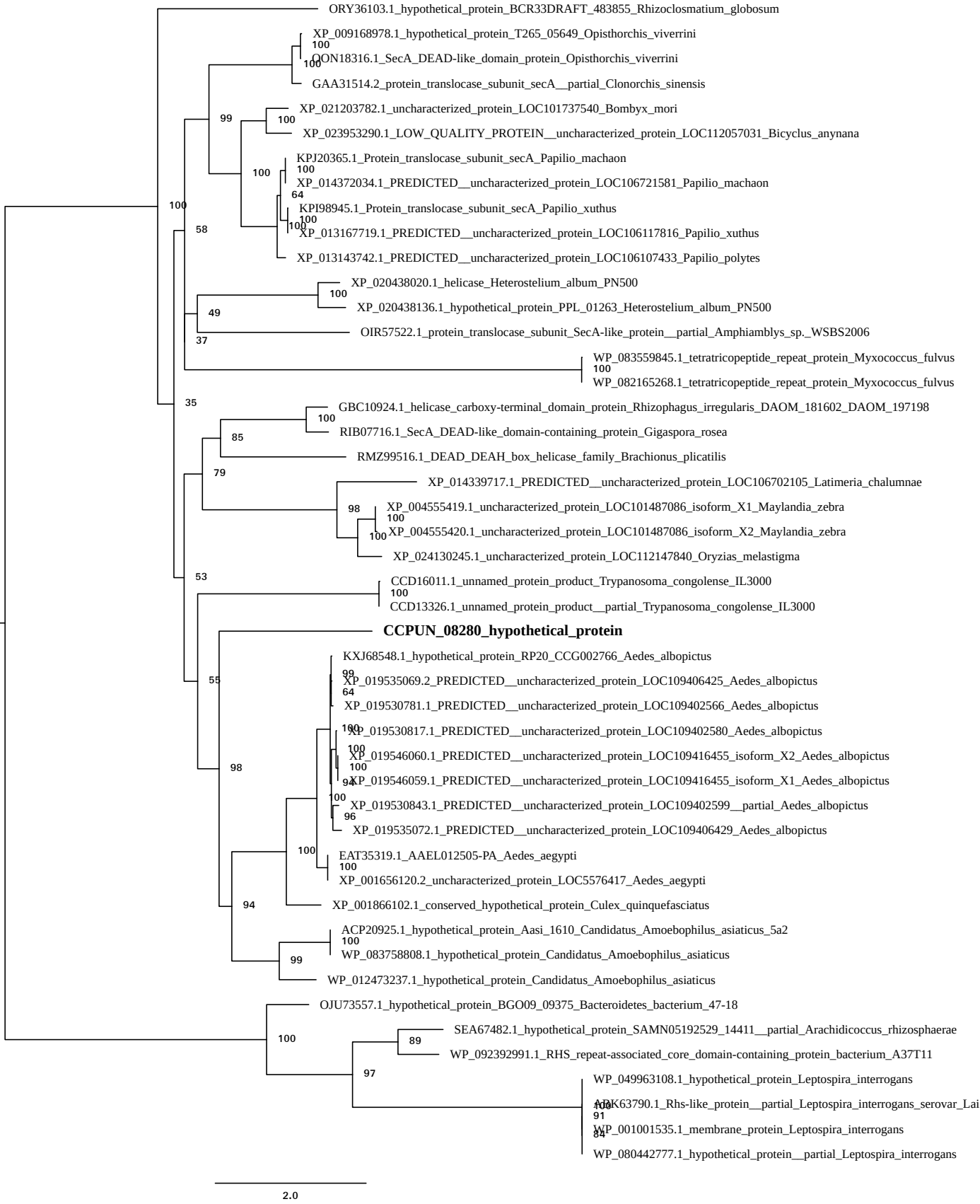

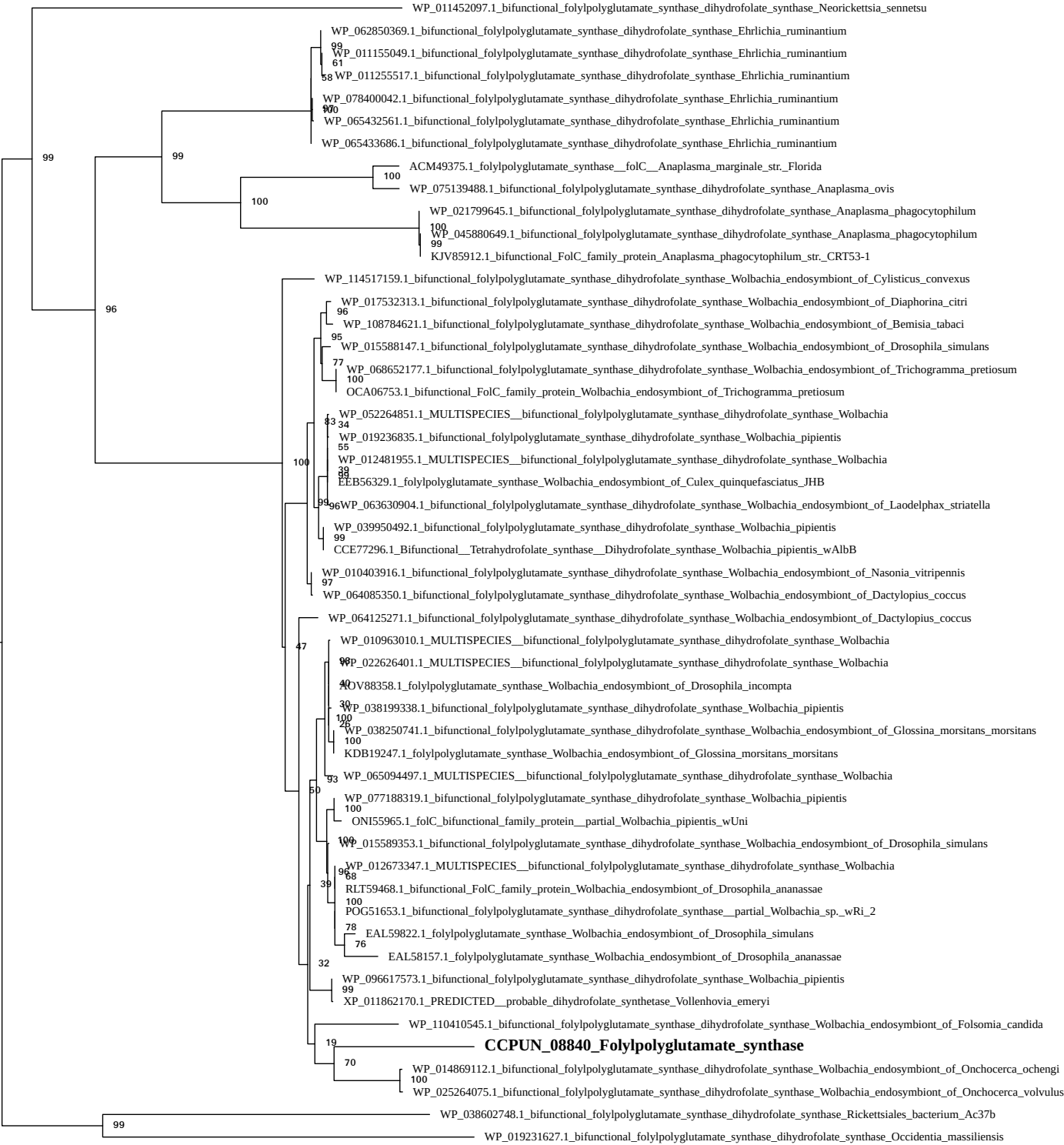

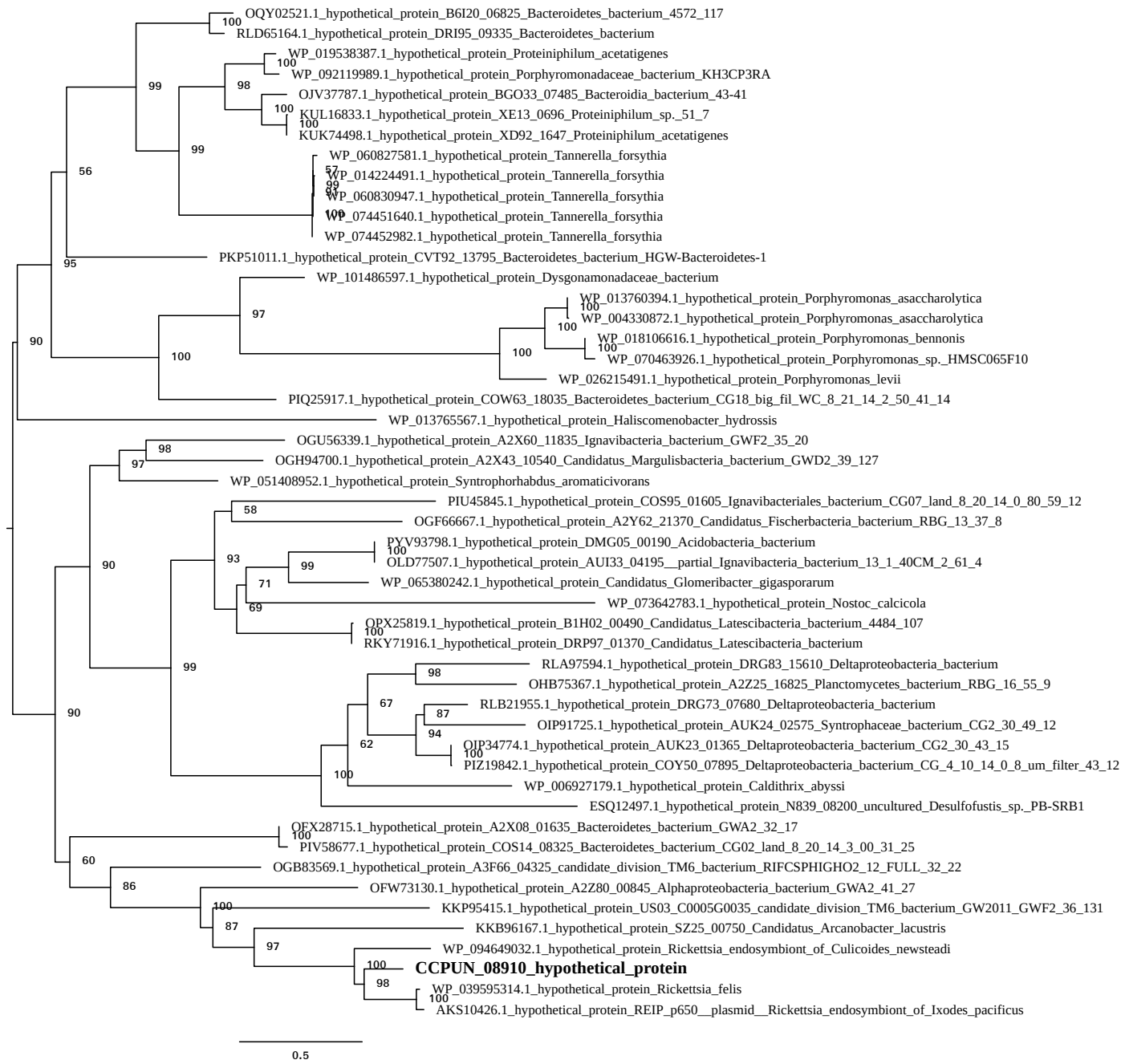

Supplement: Supplemental Information 7 — Phylogenetic relationships of cCpun HGTs with their closest homologs in the Genbank database was inferred using maximum likelihood with IQ-TREE v1.6.6 (method: automated best model selection). Branch support values are based on 1000 bootstrap replicates. [file peerj-07-6448-s007.pdf]

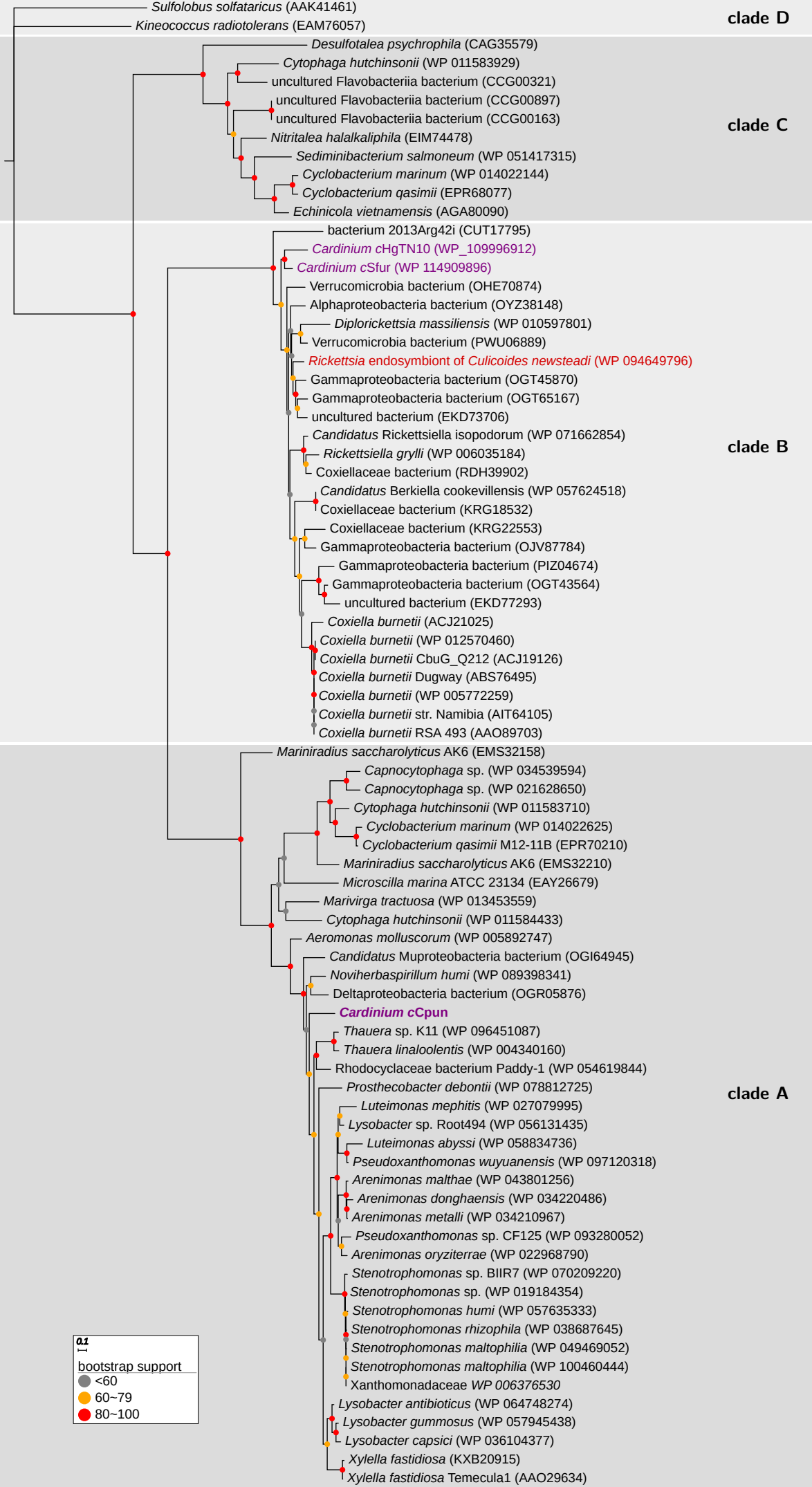

Supplement: Supplemental Information 8 — Maximum likelihood phylogenetic placement of cCpun, cSfur and cHgTN10 carbonic anhydrase (CAs) protein sequences compared with their closest homologs in the Genbank database. Members from the four clades forming the beta-class of CAs are presented. The positions of the Cardinium homologs and the CA homolog identified in Rickettsia (RiCNE) endosymbionds in biting midges are indicated in purple and red respectively. Phylogenetic relationships were inferred using IQ-TREE v1.6.6 (method: automated best model selection). [file peerj-07-6448-s008.pdf]
