# Supplementary material for: The draft genome of strain cCpun from biting midges confirms insect Cardinium are not a monophyletic group and reveals a novel gene family expansion in a symbiont
Source: PeerJ. 2019 Feb 21;7:e6448. doi: 10.7717/peerj.6448 (PMC6387759; doi:10.7717/peerj.6448)
Supplement: Supplemental Information 2 — To enhance visualization the scaffolds were size sorted and concatenated into a pseudomolecule. The alternating grey and white strips highlight the scaffold borders. Inwards, the first, second and third circle are colour coded according to COG functional categories and represent a) the complete cCpun protein coding genes, b) the core genes between the four Amoebophilaceae genomes presented in Figure 1A, and c) the cCpun unique genes. In the fourth circle we show the genomic location of the genes coding for the Afp-like (magenta) and type IX (red) secretion systems as well as the DUF1703 gene paralogs. Finally, the two line plots represent genome coverage and GC% content across cCpun genome (1kb sliding window) respectively. An orange line indicates the mean coverage (90X) of the draft assembly. [file peerj-07-6448-s002.pdf]

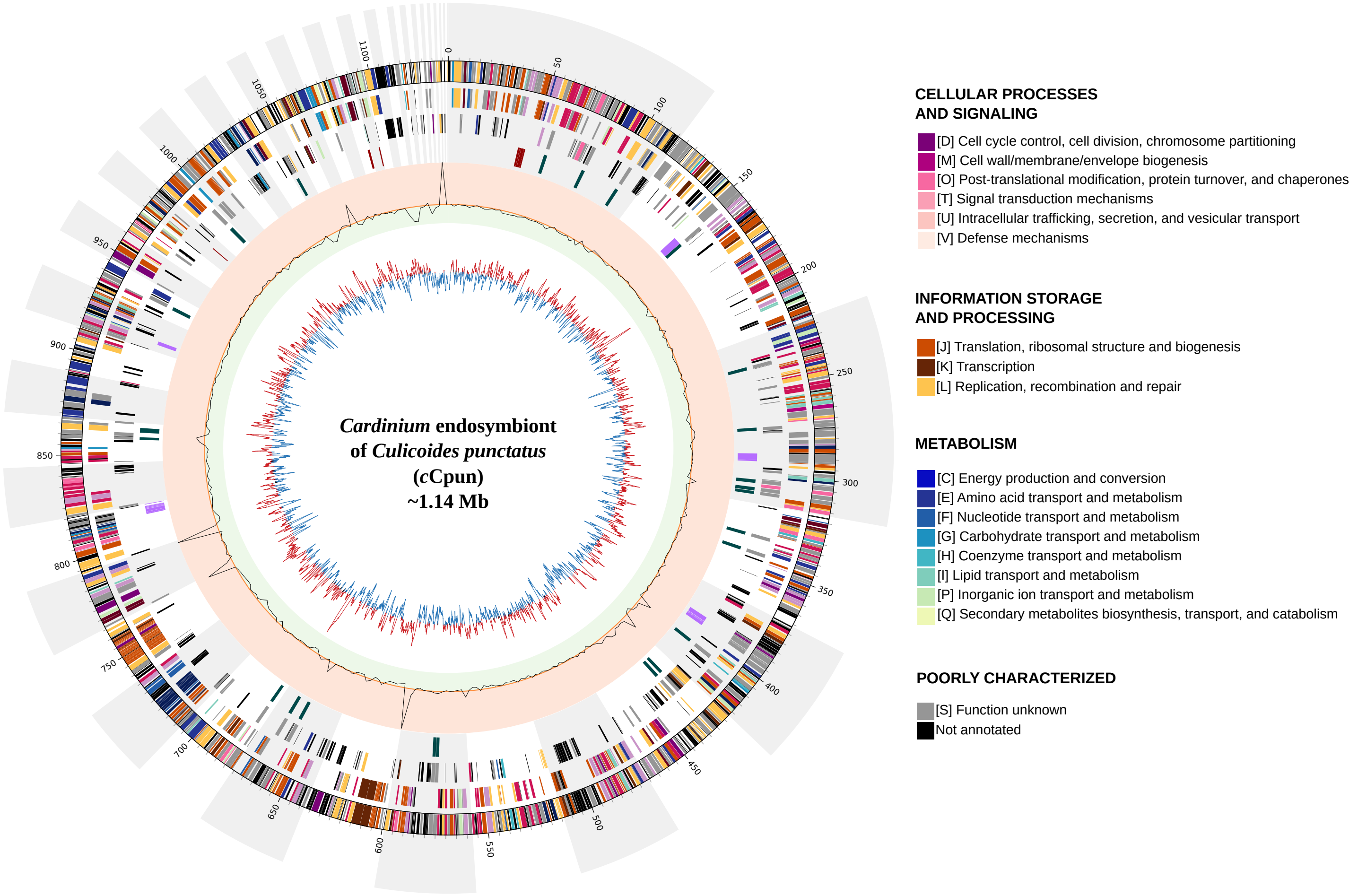

Cardinium endosymbiont of *Culicoides punctatus* (*c*Cpun)
~1.14 Mb
0
50
100
150
200
250
300
350
400
450
500
550
600
650
700
750
800
850
900
950
1000
1050
1100
CELLULAR PROCESSES AND SIGNALING
[D] Cell cycle control, cell division, chromosome partitioning
[M] Cell wall/membrane/envelope biogenesis
[O] Post-translational modification, protein turnover, and chaperones
[T] Signal transduction mechanisms
[U] Intracellular trafficking, secretion, and vesicular transport
[V] Defense mechanisms
INFORMATION STORAGE AND PROCESSING
[J] Translation, ribosomal structure and biogenesis
[K] Transcription
[L] Replication, recombination and repair
METABOLISM
[C] Energy production and conversion
[E] Amino acid transport and metabolism
[F] Nucleotide transport and metabolism
[G] Carbohydrate transport and metabolism
[H] Coenzyme transport and metabolism
[I] Lipid transport and metabolism
[P] Inorganic ion transport and metabolism
[Q] Secondary metabolites biosynthesis, transport, and catabolism
POORLY CHARACTERIZED
[S] Function unknown
Not annotated
