# Supplementary material for: The draft genome of strain cCpun from biting midges confirms insect Cardinium are not a monophyletic group and reveals a novel gene family expansion in a symbiont
Source: PeerJ. 2019 Feb 21;7:e6448. doi: 10.7717/peerj.6448 (PMC6387759; doi:10.7717/peerj.6448)
Supplement: Supplemental Information 3 — BUSCO completeness assessment results for cCpun draft genome in comparison to the other Cardinium genomes and A. asiaticus. The Results are based on the presence or absence of 148 single-copy universal bacterial markers. [file peerj-07-6448-s003.pdf]

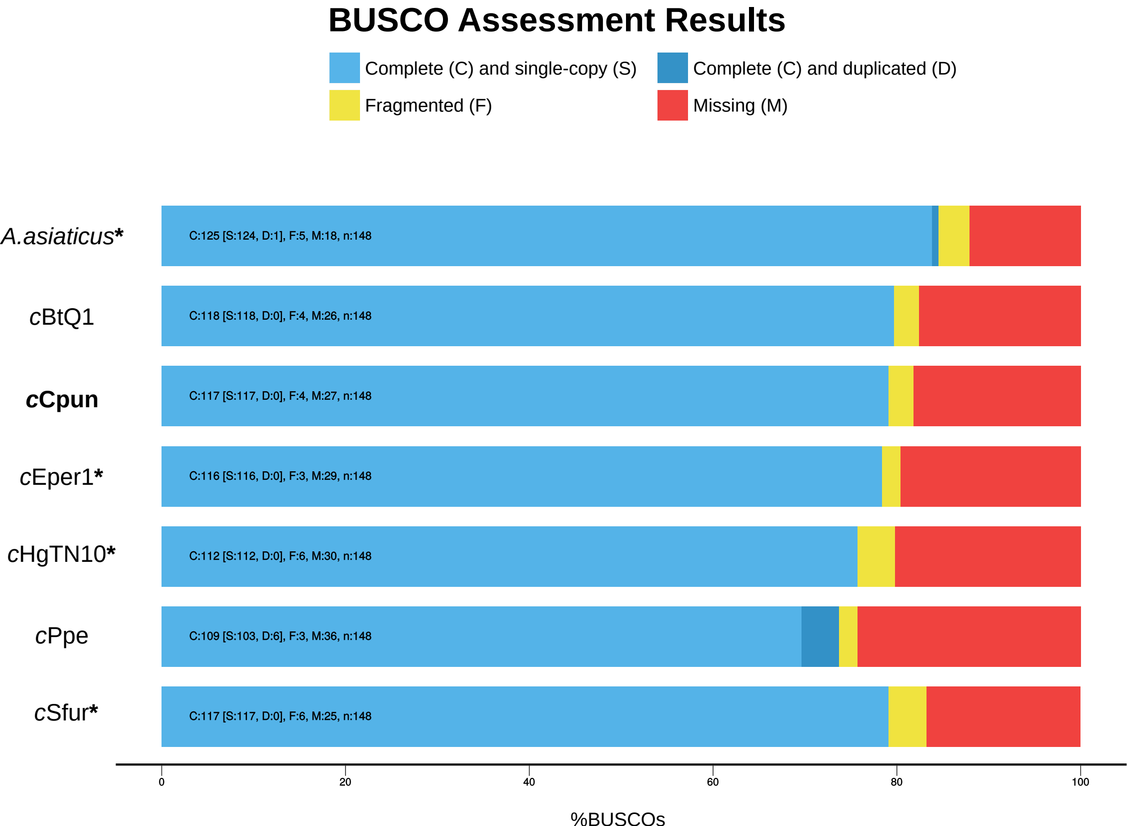
BUSCO Assessment Results
Complete (C) and single-copy (S)
Complete (C) and duplicated (D)
Fragmented (F)
Missing (M)
A.asiaticus*
C:125 [S:124, D:1], F:5, M:18, n:148
cBtQ1
C:118 [S:118, D:0], F:4, M:26, n:148
cCpun
C:117 [S:117, D:0], F:4, M:27, n:148
cEper1*
C:116 [S:116, D:0], F:3, M:29, n:148
cHgTN10*
C:112 [S:112, D:0], F:6, M:30, n:148
cPpe
C:109 [S:103, D:6], F:3, M:36, n:148
cSfur*
C:117 [S:117, D:0], F:6, M:25, n:148
0
20
40
60
80
100
%BUSCOs
