# Supplementary material for: The draft genome of strain cCpun from biting midges confirms insect Cardinium are not a monophyletic group and reveals a novel gene family expansion in a symbiont
Source: PeerJ. 2019 Feb 21;7:e6448. doi: 10.7717/peerj.6448 (PMC6387759; doi:10.7717/peerj.6448)
Supplement: Supplemental Information 6 — a) Multiple sequence alignment of the DUF1703 nuclease domain from Cardinium (blue outline) and Rickettsia (red outline) homologs described in Fig. 4. The core PD-(D/E)XK site is highlighted in yellow and the catalytic Asp, Glu and Lys residues are indicated with an asterisk. b) Residue conservation within the core PD-(D/E)XK site between the DUF1703 nuclease family depicted in (a) and the type II-IV CI-like toxins of Wolbachia and related proteins described in Gillespie et al. (2018). Sequence logos were prepared using the Skylign tool (Wheeler, Clements & Finn, 2014). [file peerj-07-6448-s006.pdf]

a)

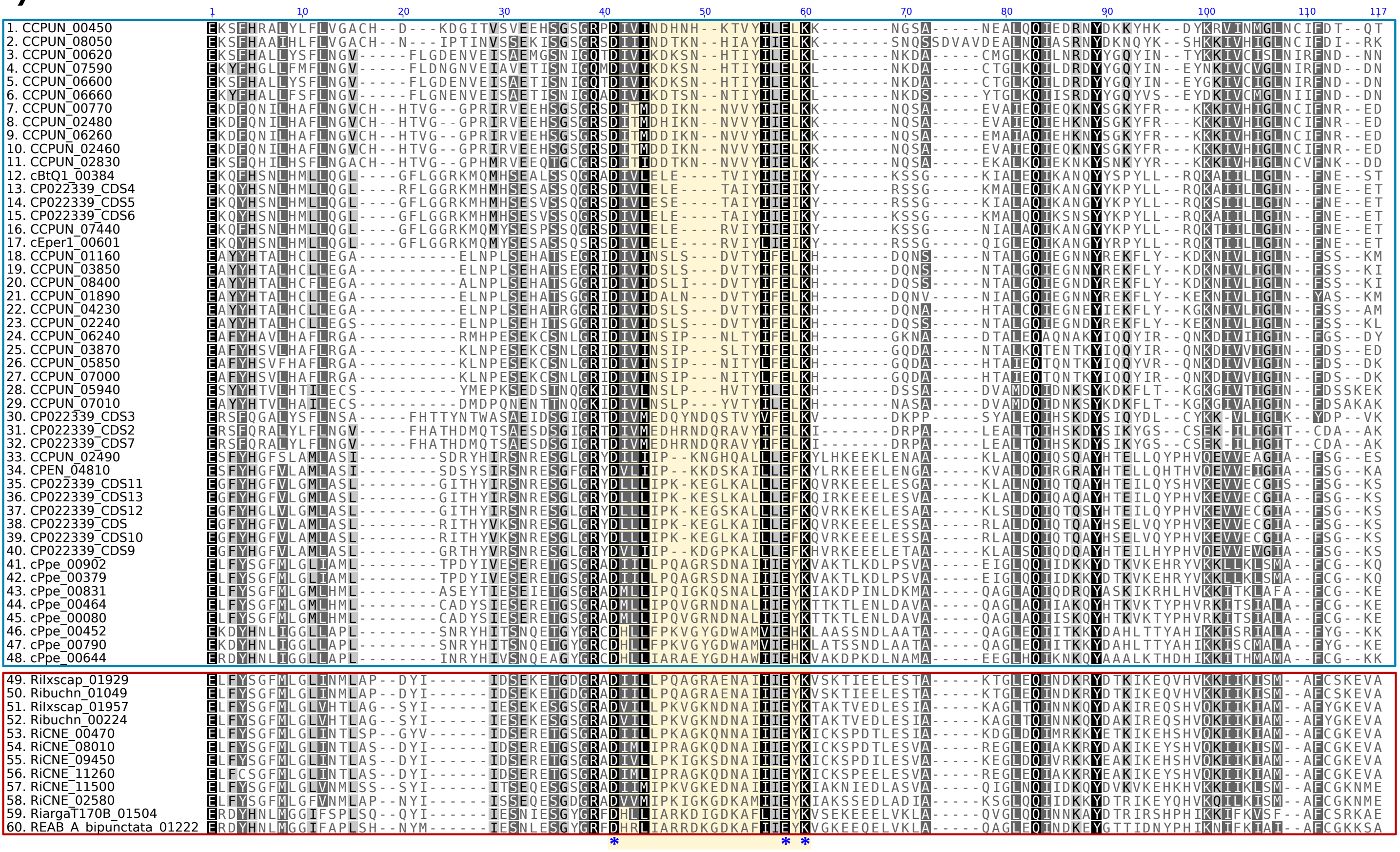

b)

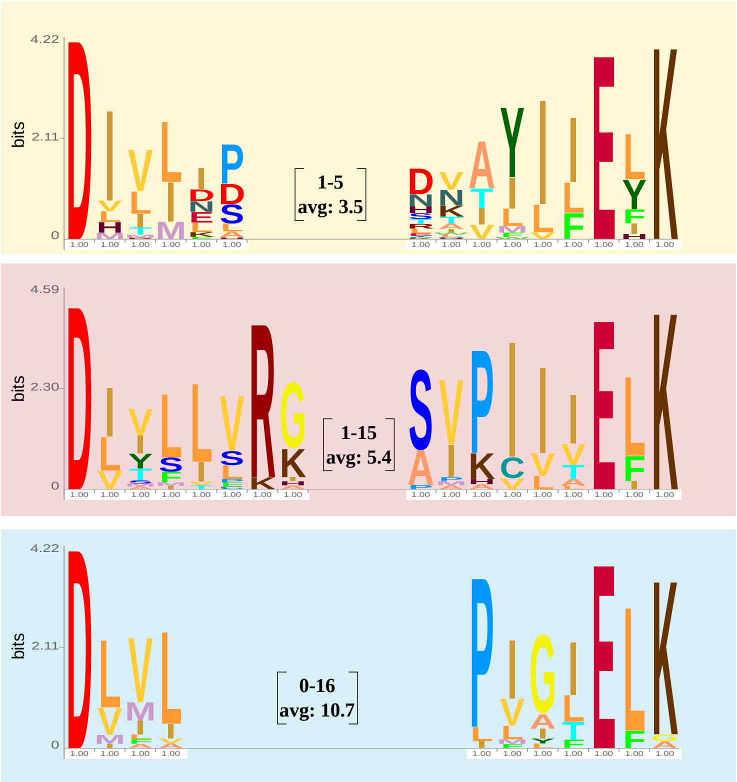

The core PD-(D/E)XK nuclease domain in the DUF1703 protein family of *Cardinium* and *Rickettsia* depicted in (a) and discribed in Figure 4.

The conserved PD-(D/E)XK nuclease domain in type II-IV CI-like toxins of *Wolbachia* and related proteins.

The cryptic PD-(D/E)XK nuclease domain in type II-IV CI-like toxins of *Wolbachia* and related proteins.
